# Drying temperatures affect the qualitative–quantitative variation of aromatic profiling in *Anethum graveolens* L. ecotypes as an industrial–medicinal–vegetable plant

**DOI:** 10.3389/fpls.2023.1137840

**Published:** 2023-05-12

**Authors:** Karim Farmanpour Kalalagh, Mehdi Mohebodini, Reza Fattahi, Arman Beyraghdar Kashkooli, Sanaz Davarpanah Dizaj, Fatemeh Salehifar, Amir Mohammad Mokhtari

**Affiliations:** ^1^ Department of Horticultural Science, Faculty of Agriculture, Tarbiat Modares University, P.O. Box 14115-336, Tehran, Iran; ^2^ Department of Horticultural Science, Faculty of Agricultural Science and Natural Resources, University of Mohaghegh Ardabili, Ardabil, Iran; ^3^ Department of Food Science and Technology, Faculty of Agriculture, Tarbiat Modares University, P. O. Box 14115-336, Tehran, Iran

**Keywords:** *Anethum graveolens*, Aromatic profiling, drying temperature, monoterpene, Sesquiterpene, α-Phellandrene

## Abstract

**Introduction:**

There are several factors that affect the quality and quantity of active ingredients and essential oil (EO) content, including pre and postharvest practices such as drying conditions. One of the most important factors in drying is temperature and then selective drying temperature (DT). In general, DT has a direct effect on the aromatic properties of *Anethum graveolens*.

**Methods:**

On this basis, the present study was conducted to evaluate the effects of different DTs on the aroma profile of *A. graveolens* ecotypes.

**Results and discussion:**

The results showed that different DTs, ecotypes, and their interaction significantly affect EO content and composition. The highest EO yield was obtained from the Parsabad ecotype (1.86%) followed by the Ardabil ecotype (1.4%), both at 40° C. More than 60 EO compounds were identified, mainly monoterpenes and sesquiterpenes, highlighting α-Phellandrene, Germacrene D, and Dill apiole as major components in all treatments. Besides α-Phellandrene, the major EO compounds at shad drying (ShD) were β-Phellandrene and p-Cymene, while plant parts dried at 40° C showed l-Limonene and Limonene as the main constituents, and Dill apiole was detected in greater amounts in the samples dried at 60 °C. To determine the appropriate DT, simple and factorial based-ANOVA together multivariate analysis demonstrated significant differences in the compounds produced under different DTs. The results indicated that more EO compounds, mainly monoterpenes, were extracted at ShD than other DTs. On the other hand, the content and composition of sesquiterpenes increased significantly when DT was increased to 60 °C. From the genetic backgrounds point of view, the Parsabad ecotype (with 12 similar compounds) and Esfahan ecotype (with 10 similar compounds) were the most suitable ecotypes under all DTs in terms of EO compounds. Accordingly, the present study would help various industries to optimize specific DT(s) to obtain special EO compound(s) from different *A. graveolens* ecotypes based on commercial requirements.

## Introduction

Dill (*Anethum graveolens* L.), an annual aromatic plant of the parsley family (*Apiaceae*, syn. *Umbelliferae*), is native to southwestern and central Asia (Naidu et al., 2016). Nowadays, it is cultivated in many parts of the world, including southeastern Europe, India, China, Pakistan, Turkey, the USA, and Iran. Its natural geographical distribution in Iran is centered in the provinces of Mashhad, Isfahan, Ardabil, Bushehr, and Kerman (Behbahani et al., 2017). The natural products of dill include essential oils (EOs), fatty acids, phenolic acids, flavonoids, etc. (Kaur and Arora, 2010). Dill EO is mainly produced in the seeds and flowers (0.2–4.6 cm^3^ 100 g^−1^), while the yield in stems and leaves is much lower (0.09**–**0.34 cm^3^ 100 g^−1^) (Callan et al., 2007). The biological activity of dill is determined by its main components in EO, namely α**-**phellandrene, germacrene D, and dill apiole (Santos et al., 2002). The profiles of these major constituents were found to vary depending on the drying temperature (DT), geographical origin, cultivar, extraction method, etc. (Pino et al., 1995b; Jirovetz et al., 2003). The relationship between the chemical composition of dill EO and its biological activities is well known and can be used as a functional additive in the pharmaceutical and food industries (Jianu et al., 2012; Naidu et al., 2016). In this regard, various functional activities of dill EO, such as antimicrobial, antioxidant, antidiabetic, and antihypercholesterolemic properties, have been demonstrated (Heamalatha et al., 2011).

Previous studies have shown that the chemical and functional properties of dill EO depend on the particular geographical region (Bowes et al., 2004). On the other hand, it has been proven that the basic step in the processing of medicinal herbs after harvest is drying. Before EO extraction, plants are dried to reduce their moisture content (Asekun et al., 2007). Some studies have shown that drying plant materials can have a significant impact on the chemical composition and functional properties of herb-derived EOs (Ahmed et al., 2018). In addition, the content of certain metabolites and bioavailability may differ between different DTs (Blanco et al., 2000). Because some of the agro**-**foods and other medicinal bioproducts, such as herbs and spices, are heat-sensitive, it is desirable to process them at optimal DTs. For example, the results of the effect of DT on the EO amount of *Mentha piperita* showed that high temperatures significantly reduced the EO content from 1% at 40°C to 0.14% and 0.12% at 60°C and 80°C, respectively. Similarly, increasing the temperature reduced the EO content of *Salvia rosmarinus* from 2.13% at 40°C to 1.62% and 1.09% at 60°C and 80°C, respectively (Khangholil and Rezaeinodehi, 2008). In another study, Mokhtarikhah et al. (2020) indicated that the maximum EO content in *Mentha spicata* was obtained by the sun-drying method. However, the maximum content of carvone as the major monoterpene was measured in the oven drying at 60°C. Ebadi et al. (2015) found that the highest EO content in *Lippia citriodora* was extracted from vacuum and oven drying samples at 60°C and 40°C, respectively. Overall, the changes in the quantity and quality of EOs during drying depend on several factors, such as the drying method and DT.

All these facts make it necessary to study the chemical composition of dill EOs from species grown in different provinces of Iran. Although there are some studies on the composition of EOs from different parts of the world, the effects of DTs on dill EOs in Iran have not been reported extensively yet. In this context, the study of the effects of the geographical origin and DTs on the content and composition of *A. graveolens* EOs is of great importance. In the present study, we aim to investigate the effects of geographical origin and DTs on the EO yield and EO quality and quantity of *A. graveolens* ecotypes. For this purpose, samples of *A. graveolens* were collected from six geographical regions of Iran, including Mashhad, Isfahan, Ardabil, Bushehr, Parsabad, and Kerman provinces. The seeds of *A. graveolens* samples were grown in the experimental field. The aerial parts of grown samples were subjected to traditional (shade drying (ShD)) and artificial (oven drying at 40°C and 60°C) DTs. Subsequently, the EO content and composition of different ecotypes were determined by gas chromatography–mass spectrometry (GC-MS).

## Materials and methods

### Experimental site

**Figure 1:**
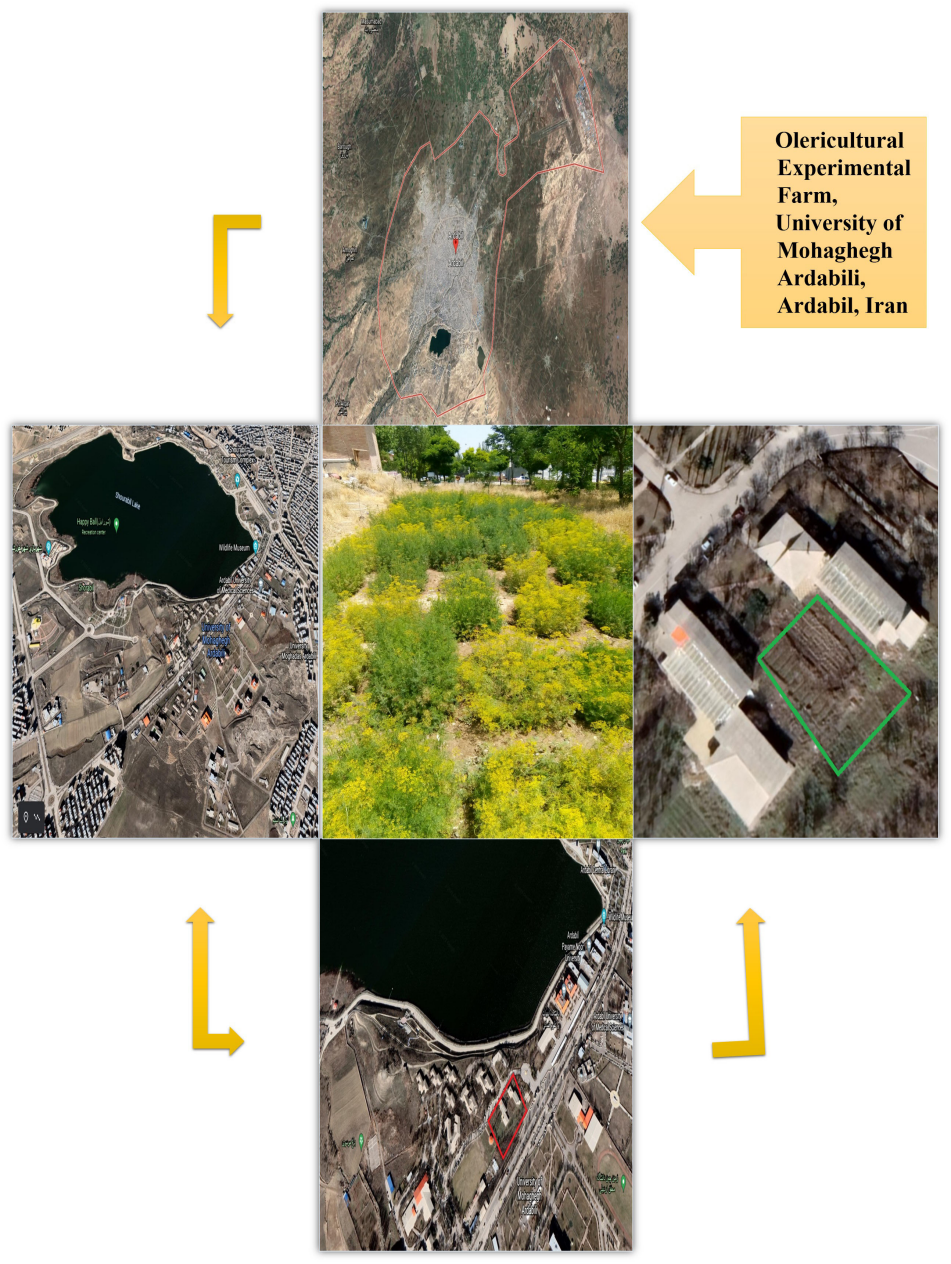
Satellite schematic location of dill (*Anethum graveolens* L.) ecotypes planting field by using "Google Earth" computer program.

The current study was conducted at the Olericulture Research Station at the University of Mohaghegh Ardabili in Ardabil province, Iran (**Figure 1**; **Table 1**). The soil properties of the field were loamy, pH = 6.85, EC = 2.38 ds/m, and Fe = 4.8 mg/kg with clay, sand, and silt contents of 13.7%, 43.2%, and 43.1%, respectively. The average annual temperature, average annual maximum temperature, average annual minimum temperature, average annual precipitation, average number of frost days, and average annual relative humidity of the experimental site were 9°C, 15.1°C, 3°C, 303.4 mm, 127 days, and 70%, respectively.

**Table 1:** Geographic location of the Iranian dill (*Anethum graveolens* L.) ecotypes collection sites.

No.	Locality	Latitude (N)	Longitude (E)	Altitude (m)
1	Ardabil	38° 15′ 13″	48° 17′ 56″	1,348
2	Mashhad	36° 15′ 56″	59° 36′ 39″	982
3	Isfahan	32° 39′ 22″	51° 40′ 19″	1,580
4	Parsabad	39° 39′ 06″	47° 55′ 13″	44
5	Bushehr	28° 54′ 43″	50° 49′ 09″	10
6	Kerman	30° 16′ 53″	57° 05′ 11″	1,764
Planting field	UMA-ORS	38° 12′ 40″	48° 17′ 37″	1,384

UMA-ORS, University of Mohaghegh Ardabili-Olericultural Research Station.

### Plant material and geographical condition of ecotypes

In our study, a total of six native dill (*Anethum graveolens* L.) ecotypes from six regions of Iran were investigated. The seeds of the ecotypes were collected separately from different regions at the full maturity stage (**Table 1**). After collecting the seeds, they were tested for viability in Plant Physiology Laboratory and selected for cultivation in the experimental field. Seeds were cultivated in three blocks and irrigated twice a week. At the stage of full flowering, the flowers were harvested together with the leaves.

### Drying temperatures

To evaluate the effect of DTs on the qualitative and quantitative variation of the aroma profile of *A. graveolens* L. ecotypes, fresh flowers and leaves were dried under three different DTs before EO extraction.

#### Shade drying

In the shade drying method, a colorless and clean cloth was flattened on the bench of the “drying room.” The fresh aerial parts of six ecotypes were then spread on the cloth in three repetitions so that no direct sunlight could reach the bench. Airflow and ventilation were controlled throughout the day under optimal conditions until the plant materials were dried.

#### Artificial drying temperatures

Fresh aerial parts from all ecotypes were scattered on the tray in three replicates. The operating temperature of the oven was set to 40°C. Finally, all dried samples were stored in special pockets and prepared for the next steps. All of the steps in this method were carried out by drying at 40°C. However, the operating temperature was adjusted at 60°C instead of 40°C.

### Extraction procedure of volatile aroma compounds

All dried samples were ground before extraction of the EOs. The 50 g of each ground sample was hydrodistillated (500 ml of distilled water) for 4 h using Clevenger apparatus. After EO extraction, the excess water was dehydrated with sodium sulfate. The purified EOs were stored in amber vials at 4°C until further analysis.

### Chromatographic analysis

The analysis of the chemical constituents of the EOs was performed using a GC-MS instrument (Agilent 5977A Series MSD). Quantification of EO compounds was performed with a GC-FID instrument (Agilent 7890 B series). Both instruments were equipped with an HP-5MS capillary column (30 m long × 0.25 mm inner diameter × 0.25 μm film thickness), and helium gas was used as the carrier gas at a flow rate of 1 ml/min in a 1:50 split ratio.

### Identification of components

The components of EO were determined using the retention time and retention index as well as NIST05 and Wiley7 mass-spectral library data. A homologous series of *n*-alkanes was used to calculate the retention indices. The constituents of EO were identified by comparing retention indices with the reference spectra database (Adams, 1995) and the National Institute of Standards and Technology (NIST) Chemistry WebBook (https://webbook.nist.gov).

### Statistical analysis

Experimental analyses were conducted based on a factorial-based randomized complete block design (RCBD) and a simple RCBD with three replicates, each separately. All data in **Table 2** were reported as the geometric means of three replicates. The main objectives of the study were as follows: (i) investigate the interaction between ecotypes and different DTs on the quantity and quality of EO profiling, (ii) separately analyze the effect of different DTs on ecotypes to determine the appropriate DT(s), and (iii) separately evaluate ecotypes at different DTs to identify superior ecotypes. The EO yield of all treatments was also listed separately in **Table 2**.

**Table 2:** Essential oils composition (% ± SE) of Iranian dill (*Anethum graveolens* L.) ecotypes under three drying temperatures (shade drying, 40°C, and 60°C).

Compound name	Formula	Classification	RI	Ardabil					Mashhad				Esfahan		
				ShD		40°C		60°C	ShD	40°C		60°C	ShD	40°C	60°C
Tricyclene	C_10_H_16_	Monoterpene	924	–		–		–	–	14.93 ± 0.106		–	–	14.79 ± 0.065	–
α-Thujene	C_10_H_16_	Monoterpene	930	0.56 ± 0.002		0.48 ± 0.002		–	0.63 ± 0.002	0.52 ± 0.004		–	0.56 ± 0.002	0.73 ± 0.004	0.22 ± 0.002
α-Pinene	C_10_H_16_	Monoterpene	939	–		2.31 ± 0.009		–	3.12 ± 0.007	2.44 ± 0.028		0.48 ± 0.07	–	3.72 ± 0.011	–
Camphene	C_10_H_16_	Monoterpene	951	0.07 ± 0.0		0.05 ± 0.0		–	0.07 ± 0.0	0.05 ± 0.0		–	0.07 ± 0.0	0.08 ± 0.0	–
1R-α-Pinene	C_10_H_16_	Monoterpene	953	3.04 ± 0.016		–		–	–	–		–	0.56 ± 0.002	–	1.37 ± 0.016
Sabinene	C_10_H_16_	Monoterpene	975	0.21 ± 0.0		0.18 ± 0.002		–	0.24 ± 0.0	0.19 ± 0.002		–	0.21 ± 0.0	0.26 ± 0.002	–
β-Pinene	C_10_H_16_	Monoterpene	979	0.29 ± 0.0		0.1 ± 0.0		–	0.22 ± 0.002	0.11 ± 0.002		–	0.22 ± 0.0	0.16 ± 0.0	–
β-Myrcene	C_10_H_16_	Monoterpene	992	0.79 ± 0.002		0.71 ± 0.004		–	0.84 ± 0.002	0.76 ± 0.009		0.19 ± 0.0	0.78 ± 0.002	0.92 ± 0.007	0.42 ± 0.004
α-Phellandrene	C_10_H_16_	Monoterpene	1,002	52.88 ± 0.096		44.54 ± 0.0		2.17 ± 0.365	44.44 ± 4.93	40.82 ± 0.098		16.43 ± 2.12	55.51 ± 0.032	43.82 ± 0.619	35.56 ± 0.245
α-Terpinene	C_10_H_16_	Monoterpene	1,017	0.11 ± 0.002		0.15 ± 0.002		–	0.13 ± 0.004	0.13 ± 0.0		–	0.12 ± 0.004	0.1 ± 0.0	0.09 ± 0.002
*p*-Cymene	C_10_H_14_	Monoterpene	1,024	9.87 ± 0.016		–		–	9.37 ± 0.021	5.08 ± 0.061		–	9.31 ± 0.014	–	–
*o*-Cymene	C_10_H_14_	Monoterpene	1,026	0.43 ± 0.002		0.7 ± 0.011		–	0.14 ± 0.0	–		–	–	12.8 ± 0.127	0.51 ± 0.014
β-Phellandrene	C_10_H_16_	Monoterpene	1,029	12.95 ± 0.023		–		–	14.47 ± 0.023	–		3.32 ± 0.417	14.07 ± 0.03	0.07 ± 0.002	7.79 ± 0.098
β-Cymene	C_10_H_14_	Monoterpene	1,030	–		–		–	–	–		1.26 ± 0.113	–	–	–
Limonene	C_10_H_16_	Monoterpene	1,032	–		22.46 ± 0.073		–	–	–		–	–	–	–
1,4-Diethylbenzene	C_10_H_14_	Ethylbenzene	1,035	–		0.04 ± 0.002		–	–	–		–	–	–	0.5 ± 0.009
*cis*-Ocimene	C_10_H_16_	Monoterpene	1,037	0.05 ± 0.0		–		–	0.04 ± 0.0	–		–	0.05 ± 0.0	–	–
*trans*-β-Ocimene	C_10_H_16_	Monoterpene	1,048	0.06 ± 0.002		–		–	–	–		–	0.04 ± 0.0	–	–
γ-Terpinene	C_10_H_16_	Monoterpene	1,059	0.06 ± 0.002		–		–	–	0.05 ± 0.0		–	0.06 ± 0.0	0.05 ± 0.002	–
α-Terpinolene	C_10_H_16_	Monoterpene	1,083	0.53 ± 0.002		–		–	–	0.22 ± 0.002		–	0.32 ± 0.0	0.25 ± 0.002	0.23 ± 0.004
1-Terpineol	C_10_H_18_O	Monoterpene	1,133	0.06 ± 0.0		0.06 ± 0.0		–	0.09 ± 0.0	0.06 ± 0.0		–	–	–	–
Sabinol	C_10_H_16_O	Monoterpene	1,149	0.08 ± 0.0		–		–	0.28 ± 0.084	–		–	0.1 ± 0.0	0.06 ± 0.0	–
Dill ether	C_10_H_16_O	Monoterpene	1,186	3.73 ± 0.009		8.03 ± 0.018		–	7.86 ± 0.028	4.69 ± 0.035		–	5.78 ± 0.002	2.79 ± 0.044	0.73 ± 0.009
Dihydrocarvone	C_10_H_16_O	Monoterpene	1,190	–		0.45 ± 0.002		–	–	0.06 ± 0.009		–	–	0.1 ± 0.004	–
Carveol	C_10_H_16_O	Monoterpene	1,224	–		–		–	–	–		0.94 ± 0.03	–	–	–
5-Hydroxymethylfurfural	C_6_H_6_O_3_	Furan	1,230	–		–		–	–	0.11 ± 0.004		–	–	–	–
D-Carvone	C_10_H_14_O	Monoterpene	1,242	–		3.69 ± 0.002		–	–	–		–	–	–	–
Carvacrol	C_10_H_14_O	Monoterpene	1,299	0.33 ± 0.004		–		–	0.3 ± 0.002	0.24 ± 0.002		0.85 ± 0.096	0.34 ± 0.0	0.4 ± 0.007	0.54 ± 0.035
Cinnamyl alcohol	C_9_H_10_O	Aromatic alcohol	1,304	0.07 ± 0.002		–		–	–	0.05 ± 0.0		–	0.32 ± 0.106	–	–
β-Damascenone	C_13_H_18_O	Monoterpene ketone	1,364	0.07 ± 0.002		–		–	–	–		–	–	–	–
β-Elemene	C_15_H_24_	Sesquiterpene	1,394	0.25 ± 0.0		–		–	–	0.07 ± 0.002		–	0.14 ± 0.0	–	–
β-Santalene	C_15_H_24_	Sesquiterpene	1,443	–		–		–	–	–		–	–	–	1.24 ± 0.016
γ-Muurolene	C_15_H_24_	Sesquiterpene	1,471	–		–		–	–	–		–	–	–	0.23 ± 0.002
γ-Gurjunene	C_15_H_24_	Sesquiterpene	1,472	–		–		–	–	–		0.56 ± 0.032	–	–	–
α-Amorphene	C_15_H_24_	Sesquiterpene	1,481	–		–		–	–	–		–	–	–	0.16 ± 0.021
Germacrene D	C_15_H_24_	Sesquiterpene	1,485	3.38 ± 0.007		0.29 ± 0.0		10.91 ± 0.176	0.81 ± 0.004	0.83 ± 0.009		5.26 ± 0.344	1.77 ± 0.002	1.32 ± 0.037	4.96 ± 0.014
β-Ionone	C_13_H_20_O	Sesquiterpene	1,488	0.17 ± 0.0		–		–	0.06 ± 0.0	–		–	–	–	0.28 ± 0.009
β-Selinene	C_15_H_24_	Sesquiterpene	1,490	0.27 ± 0.0		–		3.59 ± 0.129	–	0.03 ± 0.002		2.98 ± 0.202	–	0.21 ± 0.002	–
Myristicin	C_11_H_12_O_3_	Phenylpropene	1,518	0.69 ± 0.004		1.72 ± 0.011		–	–	1.23 ± 0.002		–	–	0.47 ± 0.014	0.56 ± 0.025
Elemicin	C_12_H_16_O_3_	Phenylpropene	1,557	–		–		–	–	0.12 ± 0.0		–	–	–	–
Germacrene B	C_15_H_24_	Sesquiterpene	1,561	0.13 ± 0.0		–		–	–	–		–	–	–	–
Dill apiole	C_12_H_14_O_4_	Phenylpropene	1,620	2.65 ± 0.004		10.84 ± 0.077		15.76 ± 0.122	3.19 ± 0.014	23.87 ± 0.117		35.3 ± 1.68	3.22 ± 0.014	12.91 ± 0.0	19.56 ± 0.124
Apiol	C_12_H_14_O_4_	Phenylpropene	1,689	–		0.14 ± 0.047		–	–	–		–	–	–	–
*m*-Diaminobenzene	C_6_H_8_N_2_	Aromatic amine	1,761	0.24 ± 0.0		–		–	–	–		–	–	–	–
Myristic acid	C_14_H_28_O_2_	Saturated fatty acid	1,776	–		–		1.23 ± 0.025	–	–		–	–	–	0.25 ± 0.0
Octadecane	C_18_H_38_	Aromatic hydrocarbon	1,802	–		–		–	–	–		–	–	–	0.06 ± 0.002
Neophytadiene	C_20_H_38_	Diterpene	1,840	0.98 ± 0.004		0.04 ± 0.0		9.05 ± 0.164	0.19 ± 0.002	0.36 ± 0.0		3.07 ± 0.115	0.45 ± 0.0	–	–
Hexahydrofarnesyl acetone	C_18_H_36_O	Sesquiterpene/Ketone	1,847	0.24 ± 0.007		–		–	0.04 ± 0.0	–		–	0.04 ± 0.0	–	0.13 ± 0.002
Dibutyl phthalate	C_16_H_22_O_4_	Phthalate ester	1,877	0.08 ± 0.00		–		8.65 ± 1.52	–	0.04 ± 0.0		–	–	0.04 ± 0.0	–
Pentadecanoic acid	C_15_H_30_O_2_	Saturated fatty acid	1,880	–		–		0.44 ± 0.016	–	–		–	–	–	–
Palmitic acid	C_16_H_32_O_2_	Saturated fatty acid	1,970	0.15 ± 0.002		0.09 ± 0.0		–	0.07 ± 0.0	0.03 ± 0.004		4.37 ± 0.292	–	0.02 ± 0.004	2.5 ± 0.304
Hexadecanoic acid, methyl ester	C_17_H_34_O_2_	Saturated fatty acid	2,005	–		–		–	–	–		–	–	–	0.11 ± 0.0
Linolenic acid, methyl ester	C_19_H_32_O_2_	Unsaturated fatty acid	2,114	–		–		–	–	–		–	–	–	0.07 ± 0.004
Linoleic acid	C_18_H_32_O_2_	Unsaturated fatty acid	2,144	–		–		0.47 ± 0.002	–	–		–	–	–	–
Diisooctyl phthalate	C_24_H_38_O_4_	Phthalate Ester	2,414	0.19 ± 0.0		–		–	–	–		–	–	–	–
Monoterpene hydrocarbons				81.9		71.68		2.17	73.71	65.3		21.68	81.88	77.75	46.69
Oxygenated monoterpenes				4.27		12.23		–	8.53	5.05		1.79	6.22	3.35	1.27
Sesquiterpene hydrocarbons				4.03		0.29		14.5	0.81	0.93		8.8	1.91	1.53	6.59
Oxygenated sesquiterpenes				0.41		–		–	0.1	–		–	0.04	–	0.41
Phenylpropenes				3.34		12.7		15.76	3.19	25.22		35.3	3.22	13.38	20.12
Diterpenes				0.98		0.04		9.05	0.19	0.36		3.07	0.45	–	–
Other				0.73		0.13		10.79	0.07	0.23		4.37	0.32	0.06	3.49
Total				95.66		97.07		52.27	86.6	97.09		75.01	94.04	96.07	78.57
**Essential oil yield (%(w/v))**				**0.1**		**1.4**		**0.12**	**0.2**	**0.21**		**0.06**	**0.22**	**0.37**	**0.1**

Compound name	Formula	Classification	RI	Parsabad					Bushehr				Kerman		
				ShD	40˚C		60˚C		ShD	40˚C	60˚C		ShD	40˚C	60˚C
Tricyclene	C_10_H_16_	Monoterpene	924	–	–		–		–	13.31 ± 0.028	–		–	–	–
α-Thujene	C_10_H_16_	Monoterpene	930	0.6 ± 0.002	0.29 ± 0.007		0.25 ± 0.002		0.56 ± 0.002	0.46 ± 0.0	0.22 ± 0.007		0.59 ± 0.007	–	0.09 ± 0.009
α-Pinene	C_10_H_16_	Monoterpene	939	3.14 ± 0.011	–		–		2.91 ± 0.004	2.72 ± 0.009	–		3.05 ± 0.031	–	0.58 ± 0.051
Camphene	C_10_H_16_	Monoterpene	951	0.07 ± 0.0	0.03 ± 0.0		0.03 ± 0.0		0.06 ± 0.002	0.05 ± 0.0	–		0.06 ± 0.002	–	–
1R-α-Pinene	C_10_H_16_	Monoterpene	953	–	1.51 ± 0.035		–		–	–	1.37 ± 0.023		–	2.48 ± 0.021	–
Sabinene	C_10_H_16_	Monoterpene	975	0.22 ± 0.0	0.12 ± 0.0		0.1 ± 0.002		0.21 ± 0.002	0.17 ± 0.0	–		0.22 ± 0.004	0.15 ± 0.002	–
β-Pinene	C_10_H_16_	Monoterpene	979	0.25 ± 0.0	0.06 ± 0.0		0.05 ± 0.0		0.2 ± 0.0	0.11 ± 0.0	–		0.21 ± 0.002	0.09 ± 0.002	–
β-Myrcene	C_10_H_16_	Monoterpene	992	0.81 ± 0.007	0.52 ± 0.009		0.45 ± 0.004		0.79 ± 0.007	0.74 ± 0.002	0.43 ± 0.009		0.81 ± 0.009	–	0.24 ± 0.009
α-Phellandrene	C_10_H_16_	Monoterpene	1,002	50.33 ± 3.57	32.46 ± 0.461		32.09 ± 0.007		54.99 ± 0.108	39.46 ± 0.134	32.16 ± 1.25		57.49 ± 0.221	47.3 ± 0.131	15.71 ± 0.975
α-Terpinene	C_10_H_16_	Monoterpene	1,017	0.17 ± 0.004	0.09 ± 0.004		0.06 ± 0.002		0.17 ± 0.002	0.08 ± 0.0	0.1 ± 0.018		0.12 ± 0.002	0.1 ± 0.0	–
*p*-Cymene	C_10_H_14_	Monoterpene	1,024	–	–		–		7.93 ± 0.047	10.29 ± 0.014	6.56 ± 0.266		8.34 ± 0.044	–	–
l-Limonene	C_10_H_16_	Monoterpene	1,027	–	26.51 ± 0.0		–		–	–	–		–	–	–
β-Phellandrene	C_10_H_16_	Monoterpene	1,029	13.3 ± 0.051	–		–		14.79 ± 0.035	–	–		14.02 ± 0.009	11.64 ± 0.03	3.44 ± 0.226
β-Cymene	C_10_H_14_	Monoterpene	1,030	5.85 ± 0.028	–		–		–	–	–		–	–	–
Limonene	C_10_H_16_	Monoterpene	1,032	–	–		26.84 ± 0.016		–	–	–		–	–	–
*cis*-Ocimene	C_10_H_16_	Monoterpene	1,037	0.04 ± 0.0	–		–		–	–	–		–	–	–
*trans*-β-Ocimene	C_10_H_16_	Monoterpene	1,048	0.08 ± 0.0	–		–		–	0.04 ± 0.0	–		0.04 ± 0.0	–	–
γ-Terpinene	C_10_H_16_	Monoterpene	1,059	0.06 ± 0.0	0.03 ± 0.0		0.03 ± 0.0		0.06 ± 0.0	–	–		0.07 ± 0.0	0.05 ± 0.0	–
α-Terpinolene	C_10_H_16_	Monoterpene	1,083	0.39 ± 0.002	0.14 ± 0.002		0.12 ± 0.0		0.26 ± 0.0	0.18 ± 0.002	–		0.33 ± 0.004	0.23 ± 0.002	–
1-Terpineol	C_10_H_18_O	Monoterpene	1,133	0.07 ± 0.002	–		–		–	–	–		0.08 ± 0.0	0.06 ± 0.004	0.12 ± 0.0
Sabinol	C_10_H_16_O	Monoterpene	1,149	–	–		–		0.1 ± 0.002	–	–		0.38 ± 0.004	–	–
Menthone	C_10_H_18_O	Monoterpene	1,151	–	–		–		–	–	0.72 ± 0.011		–	–	–
Menthol	C_10_H_20_O	Monoterpene	1,159	–	–		–		–	–	4.01 ± 0.103		–	–	–
Dill ether	C_10_H_16_O	Monoterpene	1,186	4.27 ± 0.014	6.04 ± 0.108		4.68 ± 0.007		8.84 ± 0.065	1.7 ± 0.002	0.37 ± 0.014		6.2 ± 0.047	2.41 ± 0.002	–
Dihydrocarvone	C_10_H_16_O	Monoterpene	1,190	–	1.09 ± 0.148		0.48 ± 0.002		–	–	–		–	–	–
Citronellol	C_10_H_20_O	Monoterpene	1,228	–	–		–		–	–	–		–	–	0.1 ± 0.004
D-Carvone	C_10_H_14_O	Monoterpene	1,242	–	9.94 ± 0.0		7.73 ± 0.007		–	–	–		–	–	–
Carvacrol	C_10_H_14_O	Monoterpene	1,299	0.2 ± 0.0	–		–		0.3 ± 0.002	0.4 ± 0.002	0.26 ± 0.018		0.29 ± 0.004	0.4 ± 0.011	1.01 ± 0.014
Cinnamyl alcohol	C_9_H_10_O	Aromatic alcohol	1,304	–	–		–		–	0.06 ± 0.002	–		–	0.63 ± 0.004	1.15 ± 0.431
β-Damascenone	C_13_H_18_O	Monoterpene ketone	1,364	0.09 ± 0.0	–		–		–	–	–		–	–	–
β-Bourbonene	C_15_H_24_	Sesquiterpene	1,382	–	–		–		–	–	0.34 ± 0.002		–	–	–
β-Elemene	C_15_H_24_	Sesquiterpene	1,394	–	–		–		–	0.09 ± 0.002	–		–	0.2 ± 0.002	–
α-Cedrene	C_15_H_24_	Sesquiterpene	1,411	–	–		–		–	–	–		–	–	0.25 ± 0.011
β-Santalene	C_15_H_24_	Sesquiterpene	1,443	–	–		–		–	–	0.11 ± 0.002		–	–	–
*trans*-β-Farnesene	C_15_H_24_	Sesquiterpene	1,474	–	–		–		–	–	0.16 ± 0.016		–	–	–
α-Amorphene	C_15_H_24_	Sesquiterpene	1,481	–	–		–		–	–	–		–	0.09 ± 0.002	–
Germacrene D	C_15_H_24_	Sesquiterpene	1,485	3.21 ± 0.0	0.13 ± 0.002		0.14 ± 0.0		0.92 ± 0.03	1.87 ± 0.007	2.94 ± 0.313		1.3 ± 0.009	2.8 ± 0.03	3.76 ± 0.134
Alloaromadendrene	C_15_H_24_	Sesquiterpene	1,487	–	–		–		–	–	–		–	–	0.16 ± 0.002
β-Ionone	C_13_H_20_O	Sesquiterpene	1,488	–	–		–		–	–	–		–	–	0.35 ± 0.007
β-Selinene	C_15_H_24_	Sesquiterpene	1,490	–	–		–		–	–	0.39 ± 0.134		–	0.12 ± 0.007	2.46 ± 0.07
β-Bisabolene	C_15_H_24_	Sesquiterpene	1,507	–	–		–		–	–	–		–	–	0.81 ± 0.044
Myristicin	C_11_H_12_O_3_	Phenylpropene	1,518	–	1.65 ± 0.035		1.01 ± 0.0		–	0.61 ± 0.002	–		0.05 ± 0.0	0.27 ± 0.0	–
Elemicin	C_12_H_16_O_3_	Phenylpropene	1,557	–	–		0.05 ± 0.0		–	0.08 ± 0.0	–		–	–	–
Spatulenol	C_15_H_24_O	Sesquiterpene	1,578	–	–		–		–	–	0.54 ± 0.044		–	–	–
Caryophyllene oxide	C_15_H_24_O	Sesquiterpene	1,581	–	–		–		–	–	0.32 ± 0.061		–	–	–
Viridiflorol	C_15_H_26_O	Sesquiterpene	1,587	–	–		–		–	–	0.7 ± 0.049		–	–	–
Dill apiole	C_12_H_14_O_4_	Phenylpropene	1,620	2.29 ± 0.004	18.48 ± 0.139		23.06 ± 0.04		3.81 ± 0.049	23.68 ± 0.098	16.08 ± 0.492		4.09 ± 0.011	17.51 ± 0.096	35.14 ± 0.08
Aromadendrene oxide-(2)	C_15_H_24_O	Sesquiterpene	1,641	–	–		–		–	–	0.15 ± 0.028		–	–	–
Apiol	C_12_H_14_O_4_	Phenylpropene	1,689	0.1 ± 0.0	–		–		–	–	–		–	–	–
Myristic acid	C_14_H_28_O_2_	Saturated fatty acid	1,776	–	–		–		–	–	–		–	–	0.4 ± 0.049
Neophytadiene	C_20_H_38_	Diterpene	1,840	1.03 ± 0.0	–		–		0.16 ± 0.002	0.58 ± 0.004	–		0.24 ± 0.0	–	5.65 ± 0.061
Hexahydrofarnesyl acetone	C_18_H_36_O	Sesquiterpene/Ketone	1,847	–	–		–		0.03 ± 0.002	–	–		0.05 ± 0.0	0.06 ± 0.0	0.31 ± 0.002
Dibutyl phthalate	C_16_H_22_O_4_	Phthalate ester	1,877	–	–		–		–	0.06 ± 0.0	–		–	0.11 ± 0.004	2.4 ± 0.188
Pentadecanoic acid	C_15_H_30_O_2_	Saturated fatty acid	1,880	–	–		–		–	–	–		–	–	0.25 ± 0.025
Palmitic acid	C_16_H_32_O_2_	Saturated fatty acid	1,970	0.14 ± 0.035	–		–		–	–	0.88 ± 0.068		–	–	–
Hexadecanoic acid, methyl ester	C_17_H_34_O_2_	Saturated fatty acid	2,005	–	–		–		–	–	–		–	–	0.17 ± 0.002
Linolenic acid, methyl ester	C_19_H_32_O_2_	Unsaturated fatty acid	2,114	–	–		–		–	–	–		–	–	0.08 ± 0.002
Linoleic acid	C_18_H_32_O_2_	Unsaturated fatty acid	2,144	–	–		–		–	–	–		–	–	0.12 ± 0.014
Diisooctyl phthalate	C_24_H_38_O_4_	Phthalate Ester	2,414	–	–		–		–	0.1	–		–	–	0.57 ± 0.084
Monoterpene hydrocarbons				75.31	61.76		60.02		82.93	67.61	40.84		85.35	62.04	20.06
Oxygenated monoterpenes				4.63	17.07		12.89		9.24	2.1	5.36		6.95	2.87	1.23
Sesquiterpene hydrocarbons				3.21	0.13		0.14		0.92	1.96	3.94		1.3	3.21	7.44
Oxygenated sesquiterpenes				–	–		–		0.03	–	1.71		0.05	0.06	0.31
Phenylpropenes				2.39	20.13		24.12		3.81	24.37	16.08		4.14	17.78	35.14
Diterpenes				1.03	–		–		0.16	0.58	–		0.24	–	5.65
Other				0.14	–		–		–	0.22	0.88		–	0.74	5.14
Total				86.71	99.09		97.17		97.09	96.84	68.81		98.03	86.7	74.97
**Essential oil yield (%(w/v))**				**0.16**	**1.86**		**0.4**		**0.77**	**0.16**	**0.06**		**0.46**	**0.36**	**0.04**

RI, retention index; ShD, shade drying; SE, standard error. -, not detected.

#### Univariate and multivariate analyses

Before all data were subjected to analysis of variance (ANOVA), the normal distribution of the residuals was tested for normality and randomness. ANOVA was performed using the Generalized Linear Model (GLM) procedure, followed by the Least Significant Difference (LSD) test for the comparison of means in the SAS 9.1 statistical package. This study was based on both factorial-based RCBD and simple RCBD, separately. For EO compounds including α-phellandrene, germacrene D, and dill apiole that were produced under all three DTs in all six ecotypes, factorial-based RCBD was used for statistical analysis. However, for other compounds that are not produced at all DTs and all ecotypes, simple RCBD was considered for analysis, depending on the purpose of different parts of the study.

Factor analysis was performed in SPSS 16 software. The Varimax method was used for the separation of factors. Factor loadings greater than 0.6 were considered significant for independent and main factors. Eigenvalues were calculated using a covariance matrix, and loading plots and dimensional scores were generated. Bartlett’s and Kaiser–Meyer–Olkin (KMO) tests also confirmed the adequacy of the factor analysis. The principal component analysis (PCA) was generated using SPSS 16 software to reduce the number of variables to a reduced number of new output variables (factors or principal components) that adequately reflected the main points of the original information. In addition, the data were classified into a multidimensional space using the last resulting variables as dimensions (Teles et al., 2013). For cluster analysis, NTSYS software version 2.2 was used to group ecotypes separately by three DTs. A discriminant function analysis (DFA) test was also performed to ensure accurate clustering of the initial dendrogram according to Nikrouz-Gharamaleki et al. (2019).

Venn diagrams (also known as logic diagrams, set diagrams, or primary diagrams) have been used to illustrate the number of similar and/or different compounds produced in different treatments and ecotypes. To illustrate the overlaps and nonoverlaps of the sets in the intersection and symmetric region(s) of the curves, the two web-based tools https://bioinformatics.psb.ugent.be/webtools/Venn/ and http://www.interactivenn.net/ (Heberle et al., 2015) were applied for three and six sets, respectively.

## Results and discussion

### Essential oil yield

**Figure 2:**
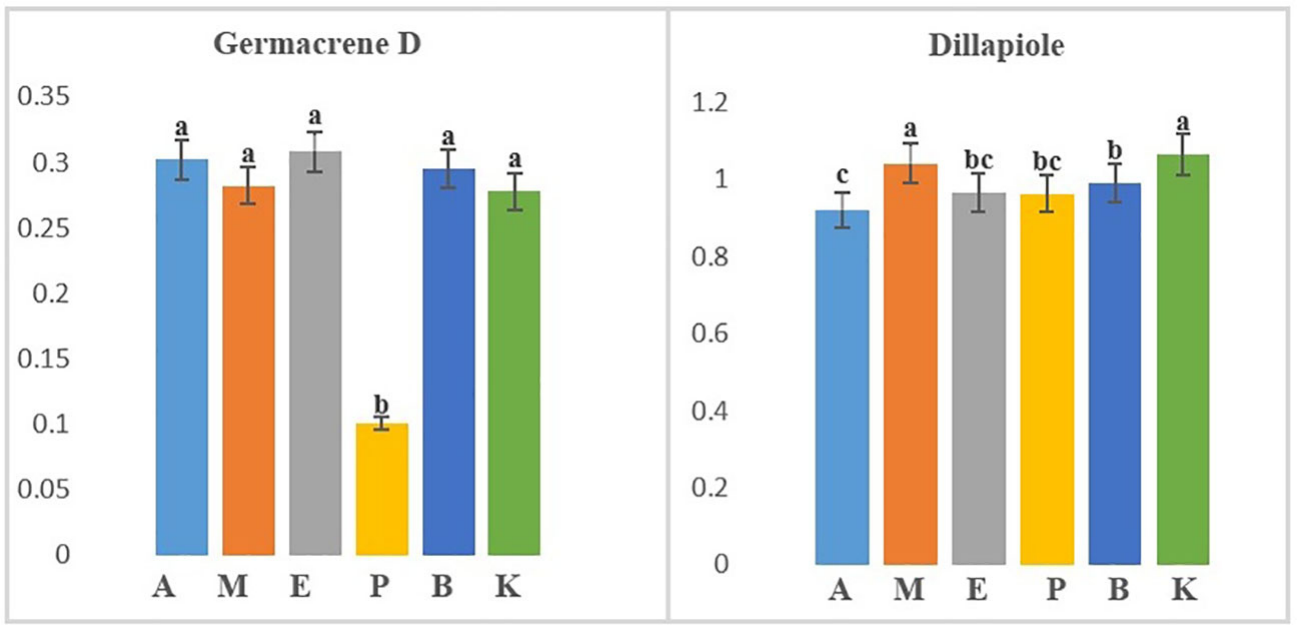
Separate mean comparison of two identified compounds in significant six dill ( *Anethum graveolens* L.) ecotypes according to 6 (ecotypes) × 3 (drying temperatures) factorial Randomized Complete Block Design (RCBD) (A, Ardabil; M, Mashhad; E, Esfahan; P, Parsabad; B, Bushehr; K, Kerman ecotypes).

The EO yield in different ecotypes depends on various factors, such as genetic background, environmental conditions, EO extraction method, organs used for EO extraction, the time required for EO extraction, postharvest DTs, etc. In this study, the EO yield (w/v %) ranged from 0.04% to 1.86% in each of the ecotypes at different DTs. So that the highest amount of EO (1.86%) was extracted in the Parsabad ecotype at 40°C and the lowest yield (0.04%) was isolated in the Kerman ecotype at 60°C (**Figure 2**). A large number of volatile compounds were identified, and their quantity and quality were different for all ecotypes at different DTs. Various volatile compounds were obtained, including terpenoids, fatty acids, and other compounds, of which monoterpenes were the most abundant compositions (**Table 2**). The amount of dill EO varied in different organs and under different climatic conditions. Studies show that the amount of EO gradually increases with the onset of flowering and reaches a maximum at flowering time. Mature seeds also have the highest amount of EO (**Supplementary Table S1**). α-Thujene, α-phellandrene, *p*-cymene, β-phellandrene, dill ether, germacrene D, and dill apiole are EO compounds from aerial parts of dill plants reported in different studies (**Supplementary Table S2**). A comparison of the production of the compounds shows that plant genetics, treatment type, planting, cultivation, harvest, and postharvesting conditions affect the quantity and quality of EO compounds. Separate isolation of each of these compounds from dill EO could have applications in various industries.

### The interaction of DTs and ecotypes (DTs × ecotypes) on EO compounds

In the study of the interaction of ecotypes and DTs (6 ecotypes × 3 DTs × 3 replicates), three volatile compounds named α-phellandrene, germacrene D, and dill apiole were produced at different levels in all treatments (**Table 2**). Results from an ANOVA based on a factorial experiment conducted in a complete block design showed no significant interaction between ecotypes and DTs. By analyzing the significant main effects, the effect of DT on the three mentioned compounds was insignificant. α-Phellandrene was also insignificant when the effect of ecotype on three compounds was examined, but germacrene D and dill apiole were significant at the 1% probability level (**Supplementary Table S3**). The mean comparison of ecotypes with respect to germacrene D showed that all ecotypes except the Parsabad ecotype were at the same level (**Figure 2**). Germacrene D is a sesquiterpene that is biosynthesized *via* the mevalonate (MVA; HMG-CoA reductase) pathway. This compound was produced in this study from 0.13% to 10.91% under 40°C DT in the Parsabad ecotype and 60°C DT in the Ardabil ecotype, respectively. Previous studies indicated that this compound was produced at 0.181% and 0.373% (Orhan et al., 2013) and 0.03%–0.18% in treatments without DT (Rostaei et al., 2018). In this study, a value of up to 10.91% was produced in the Ardabil ecotype under DT treatment. In contrast, the mean comparison of dill apiole indicates that the Kerman and Mashhad ecotypes had higher values than other ecotypes (**Figure 2**). Both ecotypes produced higher amounts of this compound at 60°C DT (35.14% and 35.3%, respectively). Dill apiole is a derivative of phenylpropene and has several applications, as mentioned earlier. In previous studies, the production of this compound in treatments without DT was reported with different values (**Supplementary Table S2**), which were up to 35.3% in our study. Overall, our results suggest that genetic background influences the biosynthesis of germacrene D and dill apiole in dill ecotypes. The prominent role of ecotypes in our ecotypes × DT results supports the findings of some previously published studies on the effects of genetic background on the quality and quantity of EO compounds. However, it is not comprehensive to draw conclusions independent of the effects of nongenetic factors on EO composition.

### Effect of DTs on ecotypes to determine the appropriate DT(s)

An analysis of variance of similar EO compounds produced in six dill ecotypes with different values at ShD showed that this temperature affected the production of compounds such as α-thujene, sabinene, β-pinene, β-myrcene, α-terpinene, β-phellandrene, dill ether, carvacrol, germacrene D, dill apiole, and neophytadiene with significance at the 0.1% probability level (**Supplementary Table S4A**). Comparison of mean (or mean difference (MD)) values of these compounds in different ecotypes shows that at ShD, α-thujene, sabinene, and β-myrcene with mean values of 0.63, 0.24, and 0.84 MD in Mashhad ecotype, β-pinene and germacrene D with mean values of 0.29 and 3.38 MD in the Ardabil ecotype, α-terpinene, β-phellandrene, and dill ether with mean values of 0.173, 14.79, and 8.84 MD in Bushehr ecotype, and carvacrol, dill apiole, and neophytadiene were highest in Isfahan (0.34 MD), Kerman (4.09 MD), and Parsabad (1.03 MD) ecotypes, respectively, compared to the other ecotypes (**Supplementary Table S5**). These results suggest that each ecotype produces a specific compound(s) when dried at ShD compared to the other ecotypes, which could be useful in selecting a particular ecotype(s) for the extraction and isolation of a specific compound(s). In this regard, previous studies have indicated that drying the aerial parts of dill at ShD resulted in the production of different amounts of the major compounds. The identified EO compounds from air-dried aerial parts of organically and conventionally grown dill showed that the α-phellandrene (27.940% and 47.748%), dill ether (9.841% and 17.344%), *p*-cymene (10.247%), β-phellandrene (7.916%), dill apiole (1.224% and 3.797%), α-thujene (0.211% and 0.287%), and germacrene D (0.181% and 0.373%) were the main compounds with the highest content (Orhan et al., 2013). On the other hand, EO compounds such as *p*-cymene (21.66%–33.66%), α-phellandrene (10.79%–34.49%), dill apiole (1.35%–12.14%), dill ether (1.47%–5.88%), germacrene D (0.03%–0.18%), and α-thujene (0.01%–0.05%) are obtained from shade-dried aerial parts of dill under the influence of chemical fertilizer and organic manure on EO composition in the sole and intercropped with *Glycine max* (Rostaei et al., 2018).

Investigation of the effects of 40°C DT in six dill ecotypes on similar EO compounds shows indicates that this DT has a significant effect on the production of compounds such as sabinene, β-pinene, α-phellandrene, α-terpinene, dill ether, germacrene D, myristicin, and dill apiole at a 0.1% probability level (**Supplementary Table S4B**). The mean comparison of these compounds in different ecotypes shows that at 40°C DT, α-terpinene (0.153 MD), dill ether (8.3 MD), and myristicin (1.723 MD) in the Ardabil ecotype, α-phellandrene (47.30 MD) and germacrene D (2.803 MD) in the Kerman ecotype, β-pinene (0.113 MD) and dill apiole (23.87 MD) in the Mashhad ecotype, and sabinene (0.203 MD) in the Isfahan ecotype have the highest values compared to other ecotypes (**Supplementary Table S5**). Therefore, these compounds are mostly produced at the highest rate in the mentioned ecotypes at 40°C DT, which may be helpful in selecting the desired ecotypes with respect to this composition. However, previous studies reported that the EO content in the dried leaves of *Piper umbellatum* L. was better at 40°C DT and air velocity of 0.4 m s^−1^ (Dorneles et al., 2019). Furthermore, convective drying of *Melissa officinalis* L. leaves at 40°C DT has increased the geranial and neral (Argyropoulos and Müller, 2014). A significant study was also conducted in the field of drying methods on the EO content of *Coriandrum sativum* L., so that at 40°C DT, *n*-decanol (13.15%) and *trans*-2-undecen-1-ol (12.88%) were produced in the highest amounts compared to other drying methods (Ghasemi Pirbalouti et al., 2017). In another successful example in *Zingiber montanum* rhizomes under different DT and drying methods, α-terpinyl acetate (0.34%), germacrene B (0.50%), (E)-1-(3′,4′-dimethoxyphenyl)but-1-ene (1.44%), (E)-1-(3′,4′-dimethoxyphenyl)buta-1,3-diene (DMPBD) (29.93%), and (E)-1-(2′,4′,5′-trimethoxyphenyl)buta-1,3diene (TMPBD) (2.99%) were identified with higher values at 40°C DT (Mahayothee et al., 2020). It is noteworthy that in addition to 40°C DT, α-phellandrene (2.50%), camphene (0.89%), E-β-ocimene (0.26%), and 1,8-cineole (63.19%) along with *trans*-2-hexenal (0.24%) are also detected higher than other compounds in *Laurus nobilis* L. leaves in the oven 45°C and infrared 45°C, respectively (Sellami et al., 2011). Moreover, the maximum production of 1,8-cineole (34.54%; in electric baking drying at 50°C) and sabinene (0.57%; in the oven drying at 50°C) is affected by the drying methods in *Amomum tsao-ko* (Qin et al., 2021). In the case of *Anethum graveolens* L., it has also been found that the concentration of Apiol (47.69%) increases at 50°C DT compared to the fresh plant (20.50%) (Naidu et al., 2016).

ANOVA of similar compounds produced with different values at 60°C DT in dill ecotypes indicates that this DT has a significant effect on the production of α-phellandrene, germacrene D, and dill apiole at 0.1% probability level (**Supplementary Table S4C**). The results of the mean comparison showed that germacrene D (10.91 MD) was highest in the Ardabil ecotype and α-phellandrene (35.56MD) and dill apiole (35.30 MD) were highest separately in the Isfahan and Mashhad ecotypes compared to other ecotypes (**Supplementary Table S5**). According to some sources dealing with different postharvest DTs, 60°C DT has also had a positive effect on the qualitative and quantitative characteristics of EO in some plant species. In this regard, the production of cadinol (2.28%), eremoligenol (1.51%), and muurol-5-em-4-β-ol (0.69%) was significantly increased at 60°C DT in *Piper umbellatum* L. leaves (Dorneles et al., 2019). Also in lemon peel, infrared drying at 60°C resulted in the production of the highest amount of d-limonene (70%), neryl propionate (1.03%), citronellyl butyrate (0.57%), and neryl acetate (0.16%) compared with other DTs (Zhang et al., 2018). For comparison, the type of drying at 60°C affected the production of some compounds in *Lippia citriodora* Kunth. In this respect, the production of γ-elemene (6.5%) and α-terpineol (1.1%) at 60°C oven drying and limonene (8.2%), sabinene (2.4%), E-caryophyllene (1.6%), Epi-α-cadinol (0.7%), and α-pinene (0.2%) at 60°C vacuum drying showed a significant increase compared to other compounds (Ebadi et al., 2015). In another successful study in terms of the type of 60°C DT, carvone (53%) and borneol (1.2%) were affected by the oven drying, while the highest amount of pulegone (12.8%) was produced by vacuum drying (Mokhtarikhah et al., 2020). Furthermore, the maximum value of 1-tetradecanol (31.74%), *cis*-phytol (34.05%) (Ghasemi Pirbalouti et al., 2017), sabinene (43.89%), α-thujene (0.31%) (Mahayothee et al., 2020), citronellol, caryophyllene oxide, and β-caryophyllene (Argyropoulos and Müller, 2014) are produced under 60°C DT.

To the best of our knowledge, more similar compounds with different values are produced at ShD in six ecotypes than other DTs. However, the quantity and quality of the compound(s) must be examined in detail in all three temperatures to determine which temperature is best for a particular compound. The results of EO compounds in our *Anethum graveolens* L. ecotypes show that sesquiterpenes were usually significantly produced at 60°C DT. Apart from DTs, other factors, such as altitude, may also influence the content of EOs. In this regard, Talebi et al. (2019a) reported that in *Nepeta* species, the content of oxygenated EO compounds increases with altitude, while the content of other monoterpenes decreases with the altitudinal gradient. Since hydrogenated and oxygenated monoterpenes and sesquiterpenes can be produced under the influence of different DTs and treatments, genetic background also affects their production (**Table 2**). EO profile of Iranian *Salvia nemorosa* L. populations also showed that sesquiterpene hydrocarbons were produced in the Tehran population (44.59%), and oxygenated sesquiterpenes were produced in high amounts in Shazand, Amir Kabir, Kerman, and Sangak populations (Mahdieh et al., 2018). Sesquiterpenes and oxygenated compounds were also detected in the Iranian *Salvia chloroleuca* population in the EO of the Neyshabur population in higher amounts than in the other populations (Talebi et al., 2019b).

Thus, the change in the production of compounds in these ecotypes could be due to the increase in DTs. On the other hand, the high production of sesquiterpenes (compared to monoterpenes) at high DTs could be related to the formation of by-products from other compounds, including monoterpenes. Unsaturated compounds may be affected by photochemical cycloaddition reactions (PCARs), which are responsible for the formation of several products. PCARs are thought to be involved in the decomposition steps of these compounds because they are polymerized by exposure to air and light (Copolovici and Niinemets, 2018).

### Ecotypes at different DTs to determine superior ecotypes

**Figure 3:**
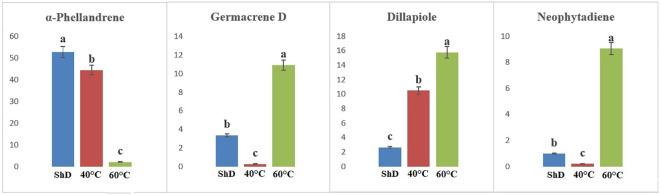
Mean comparison of significant compounds in Ardabil dill ( *Anethum graveolens* L.) ecotypes under three drying temperatures (shade drying (ShD), 40°C, and 60°C).

**Figure 4:**
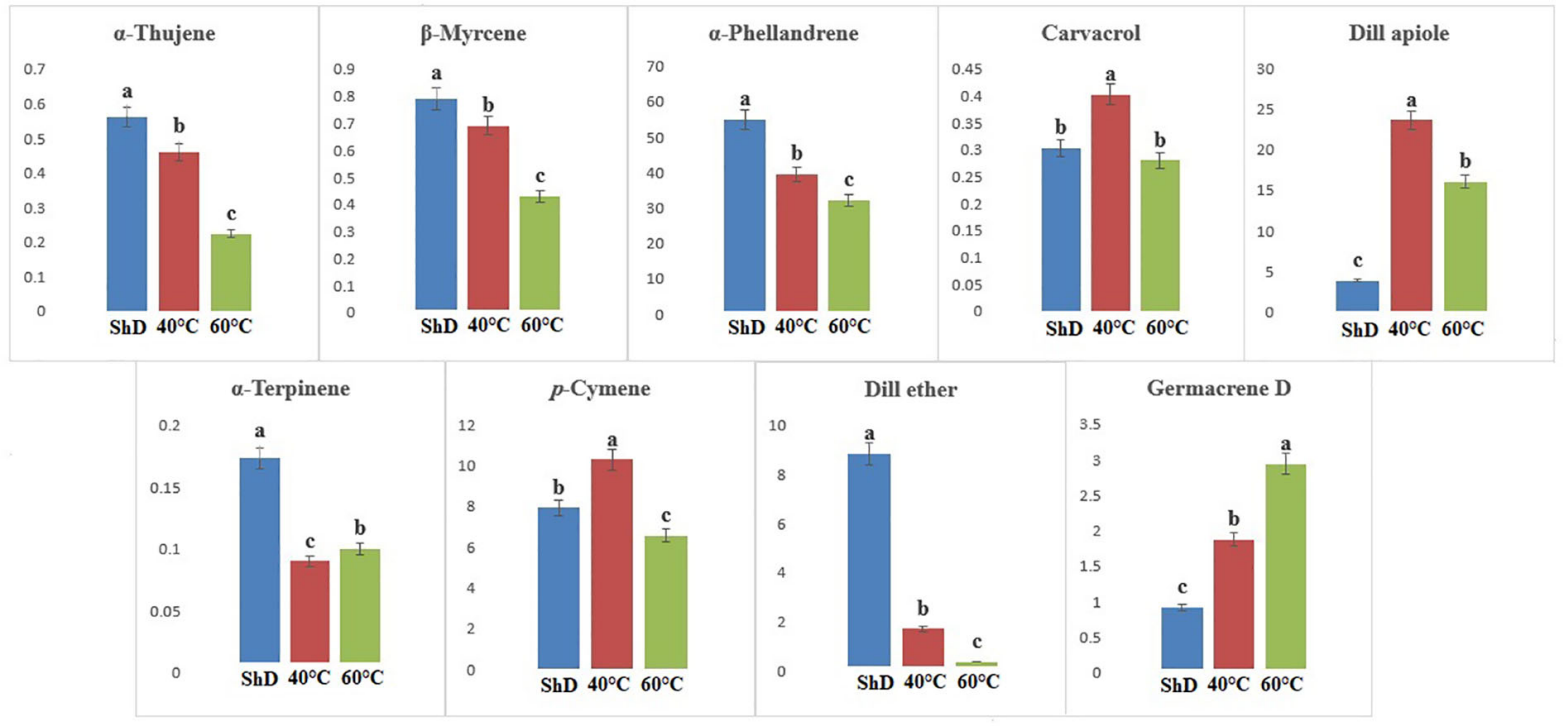
Mean comparison of significant compounds in Bushehr dill (*Anethum graveolens* L.) ecotypes under three drying temperatures (shade drying (ShD), 40°C, and 60°C).

**Figure 5:**
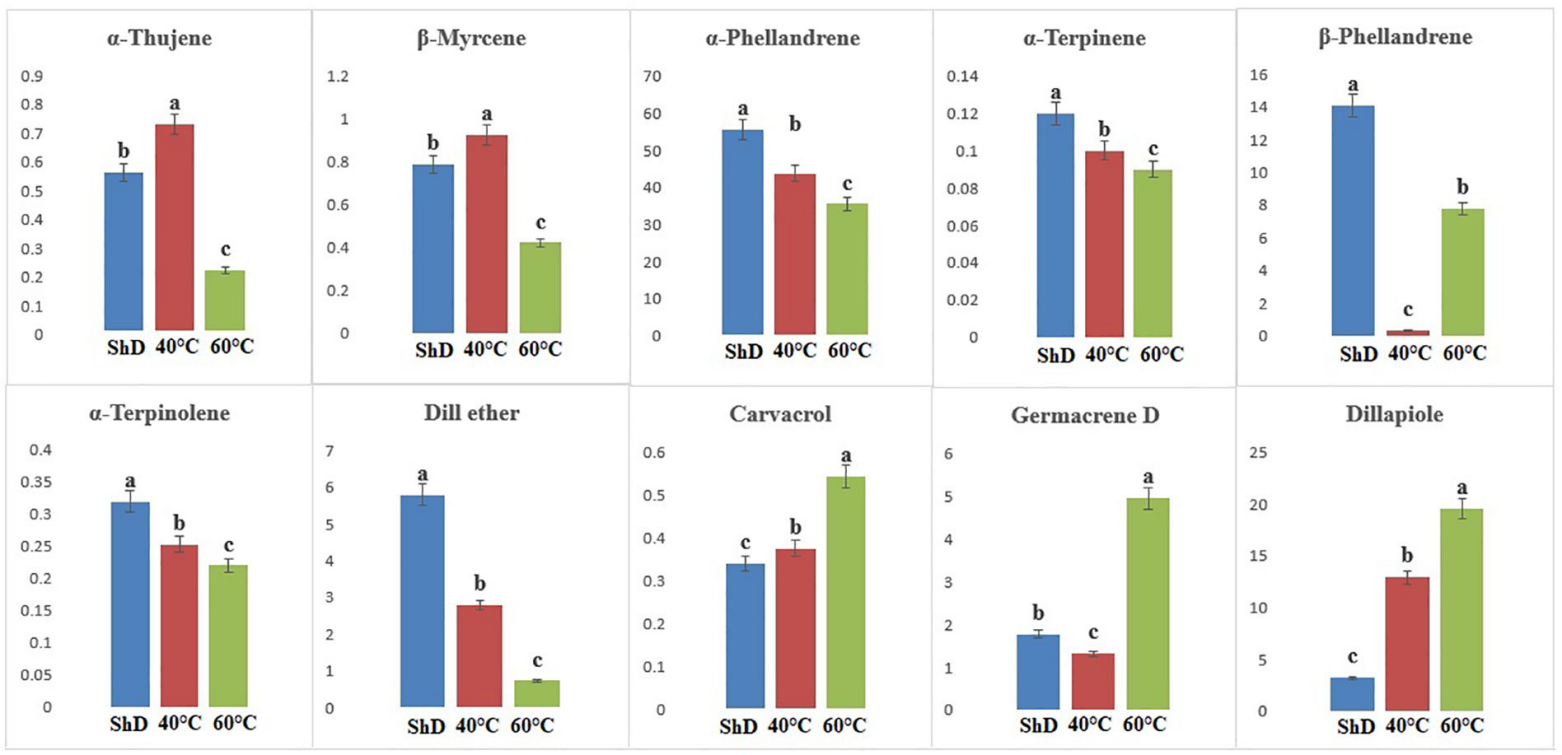
Mean comparison of significant compounds in Esfahan dill (*Anethum graveolens* L.) ecotypes under three drying temperatures (shade drying (ShD), 40°C, and 60°C).

ANOVA of similarly produced EO compounds in the Ardabil ecotype at three different DTs shows that α-phellandrene, germacrene D, dill apiole, and neophytadiene are significant at the 0.1% probability level (**Supplementary Table S6A**). A mean comparison of these compounds demonstrates that α-phellandrene at ShD and germacrene D, dill apiole, and neophytadiene at 60°C is at the best level compared to other DTs (**Figure 3**). These results indicate that the 60°C DT is suitable for the production of the four mentioned compounds in the Ardabil ecotype. In the Bushehr ecotype, 12 similar compounds were produced under different DTs, including α-terpinene (at 5% probability level), *p*-cymene, carvacrol, and germacrene D (at a 1% probability level), and α-thujene, β-myrcene, α-phellandrene, dill ether, and dill apiole (at 0.1% probability level) (**Supplementary Table S6B**) with different values. The mean comparison of these compounds indicates that ShD for α-thujene, β-myrcene, α-phellandrene, α-terpinene, and dill ether, 40°C for carvacrol, dill apiole, and *p*-cymene, and 60°C is the most suitable DT for germacrene D in this ecotype (**Figure 4**). Three different DTs in the Isfahan ecotype resulted in the production of 13 similar compounds with different values at all temperatures. In this ecotype, α-thujene, β-myrcene, α-phellandrene, β-phellandrene, α-terpinolene, dill ether, germacrene D, and dill apiole at 0.1% probability level, α-terpinene at 1% probability level, and carvacrol at 5% probability level showed a significant production (**Supplementary Table S6C**). The mean comparison of these compounds proves that the ShD for α-phellandrene, α-terpinene, β-phellandrene, α-terpinolene, and dill ether, 40°C for α-thujene and β-myrcene, and 60°C for carvacrol, germacrene D, and dill apiole are the best DTs (**Figure 5**).

**Figure 6:**
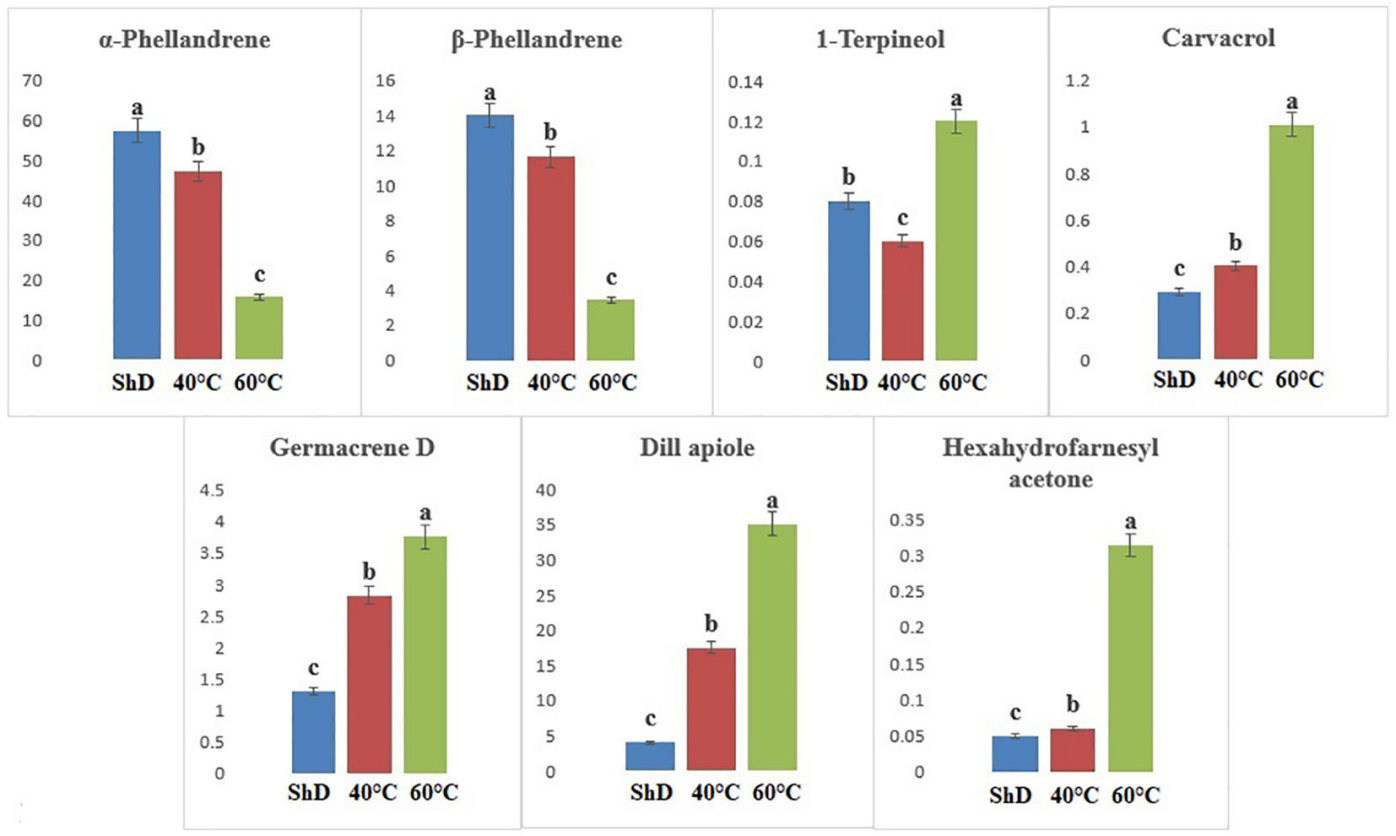
Mean comparison of significant compounds in Kerman dill (*Anethum graveolens* L.) ecotypes under three drying temperatures (shade drying (ShD), 40°C, and 60°C).

**Figure 7:**
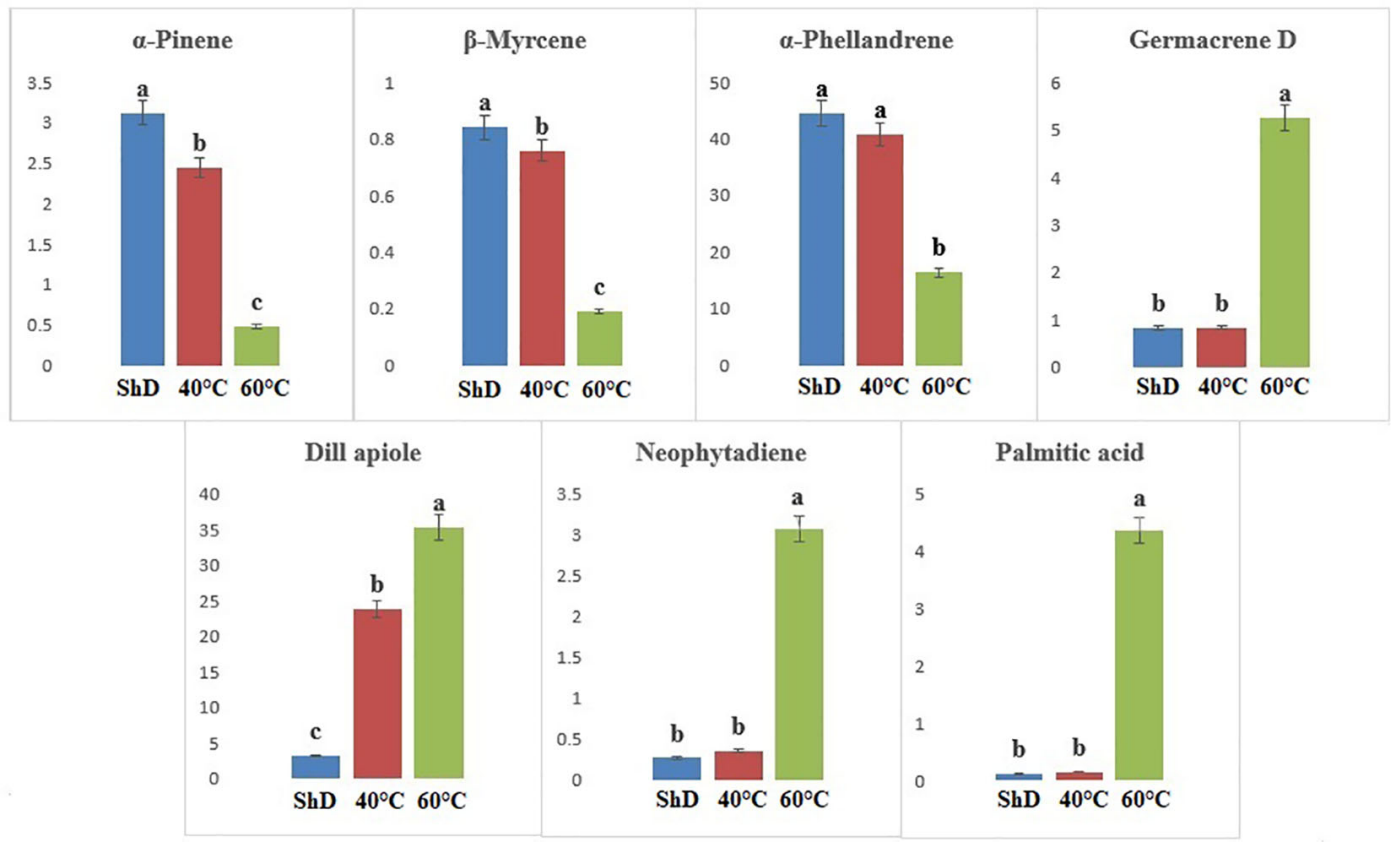
Mean comparison of significant compounds in Mashhad dill (*Anethum graveolens* L.) ecotypes under three drying temperatures (shade drying (ShD), 40°C, and 60°C).

According to ANOVA, the importance of compounds such as 1-terpineol (at 1% probability level) and α-phellandrene, β-phellandrene, carvacrol, germacrene D, dill apiole, and hexahydrofarnesyl acetone (at 0.1% probability level) in the Kerman ecotype is greater than other compounds (**Supplementary Table S6D**). Thus, the production of 1-terpineol, carvacrol, germacrene D, dill apiole, and hexahydrofarnesyl acetone at 60°C and the α-phellandrene and β-phellandrene at ShD is better than other DTs (**Figure 6**). Different DTs in the Mashhad ecotype resulted in the production of α-pinene, β-myrcene, α-phellandrene, germacrene D, dill apiole, neophytadiene, and palmitic acid with different values for all DTs. Except for α-phellandrene, which was significant at a 1% probability level, the other produced compounds were significant at a 0.1% probability level (**Supplementary Table S6E**). ShD was considered the most suitable DT for α-pinene, β-myrcene, and α-phellandrene and 60°C for germacrene D, dill apiole, neophytadiene, and palmitic acid (**Figure 7**). Since ShD was the best DT for α-pinene production in this study, α-pinene was also identified as the major EO constituent of all populations (15.5%–25.35%) at room temperature in wild populations of *Salvia multicaulis* Vahl (Talebi et al., 2021).

**Figure 8:**
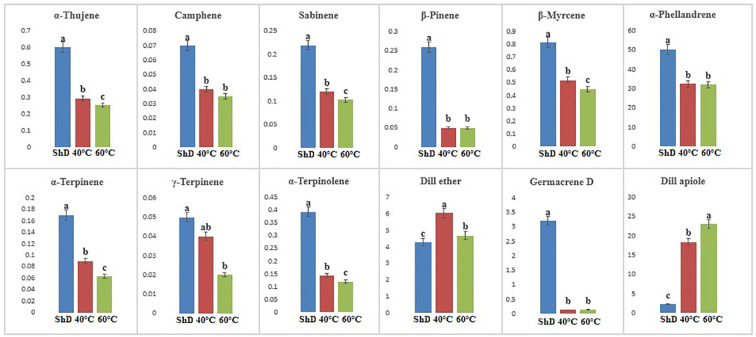
Mean comparison of significant compounds in Parsabad dill (*Anethum graveolens* L.) ecotypes under three drying temperatures (shade drying (ShD), 40°C, and 60°C).

In the Parsabad ecotype, 12 similar compounds with different values were produced. α-Thujene, sabinene, β-pinene, β-myrcene, α-terpinene, α-terpinolene, dill ether, germacrene D, and dill apiole at 0.1% probability level, camphene and α-phellandrene at 1% probability level, and γ-terpinene had significant production at the 5% probability level (**Supplementary Table S6F**). The mean comparison of the significant compounds shows that the 60°C for dill apiole, 40°C for dill ether, and ShD for α-thujene, sabinene, β-pinene, β-myrcene, α-terpinene, α-terpinolene, germacrene D, camphene, α-phellandrene, and γ-terpinene are the optimal DTs (**Figure 8**).

Altogether, *A. graveolens* L. ecotypes differed significantly in the quantity and quality of produced EO compounds. Therefore, in addition to exogenous factors (environmental conditions, EO extraction method, time required for EO extraction, postharvest treatments, etc.), other endogenous factors (plant age, EO production, and accumulation site, genetic factors regulating the biosynthesis of secondary metabolite, etc.) may influence the qualitative and quantitative content of EO (Barra, 2009; Franz and Novak, 2009). Some processes leading to the evolution and development of plant volatile formation are (i) gene amplification (gene duplication or chromosomal duplication) after divergence, in which the basic enzymatic activity is preserved while a new activity evolves from the amplified gene; (ii) convergent evolution, in which new functions have arisen independently several times; (iii) evolution of an existing gene without duplication, in which a new enzymatic activity arises from the loss of the original gene; (iv) enzymatic dysfunction due to chromosomal rearrangements/mutations, hybrid formation; (v) etc.

In all of the cases mentioned, each of these processes results in changes in gene expression patterns. In addition, very small changes in enzyme structure lead to functional enzymatic variety, so exposure to fluctuating environments leads to an increase in this diversity. On the other hand, a change in protein expression may not deactivate enzymatic activity, but it may lead to the production of secondary metabolites in different cells, tissues, or organs (Gang, 2005; Figueiredo et al., 2008). Taken as a whole, considering genetic background according to our results, the Isfahan ecotype (with 13 similar compounds) and Bushehr, together with the Parsabad ecotype (with 12 similar compounds), were the most suitable ecotypes in all three DTs in terms of volatile quality. However, the choice of an ecotype in terms of a particular compound(s) depends on the objectives of the researchers and the requirements of the industry.

### Factor analysis, cluster dendrogram, and Venn plots

Factor analysis based on Varimax rotation in DTs resulted in the generation of four factors with 13 effective compounds at ShD. The one to four factors explained 38.973%, 25.111%, 14.578%, and 12.501% of the variance and 91.163% of the cumulative variance, respectively. β-Pinene, β-phellandrene, dill ether, germacrene D, dill apiole, and neophytadiene in the first factor, α-thujene, sabinene, β-myrcene, and α-phellandrene in the second factor, camphene in the third factor, and α-terpinene and carvacrol in the fourth factor with loading values ≥ 0.6 played the largest role in the extraction of each factor. At 40°C, three factors with eight effective compounds were determined with variances of 42.925%, 27.728%, and 21.173% and a cumulative variance of 91.826%, respectively. The α-terpinene, dill ether, germacrene D, and myristicin in the first factor, sabinene and β-pinene in the second factor, and α-phellandrene and dill apiole in the third factor showed the most important role. At 60°C, two factors with three effective compounds were produced. The percentage of variance in the first and second factors was 62.669% and 31.161%, respectively, resulting in 93.830% of cumulative variance. α-Phellandrene and germacrene D in the first factor and dill apiole in the second factor with high loading values indicated the most significant role in the extraction of each factor (**Supplementary Table S7**).

**Figure 9:**
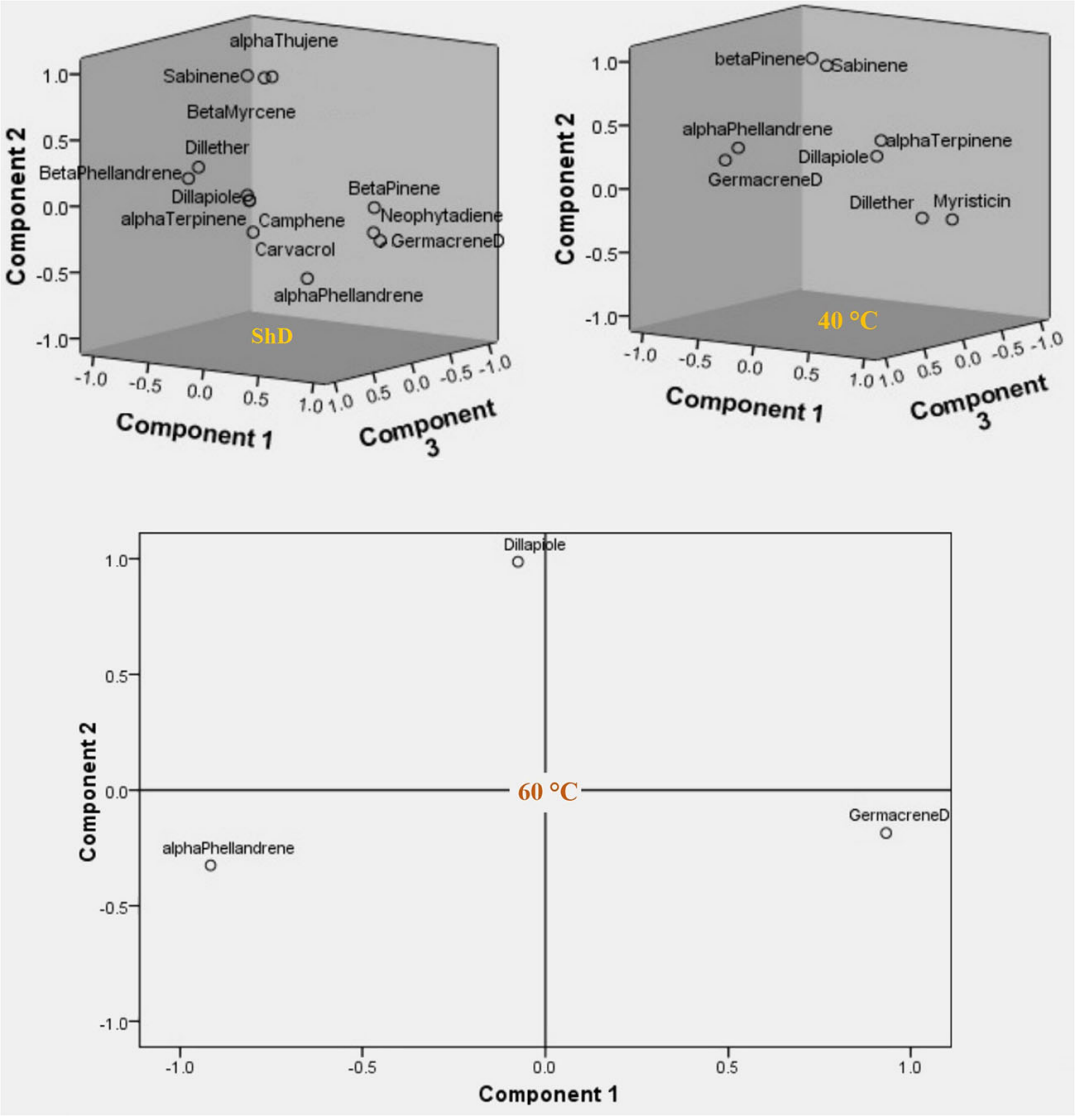
Component plots in rotated space for evaluating of three drying temperatures (shade drying (ShD), 40°C, and 60°C) on dill (*Anethum graveolens* L.) ecotypes.

The specific/and or total value of each factor is the ratio of the variance of the total variable explained by that factor. A low value means that this factor plays a minor role in explaining the variance of the variables. On the other hand, the number of factors extracted in each of the DTs means that at ShD, 40°C, and 60°C with four, three, and two factors, respectively, make the greatest contribution to explaining the variance of the data. In this context, the three-dimensional (ShD and 40°C) and two-dimensional (60°C) diagrams of the variables in relation to the extracted factors show the distribution for the studied variables in relation to the first, second, and third factors at ShD along with 40°C and the first and second factors at 60°C (**Figure 9**). On the other hand, the factor analysis according to **Supplementary Table S8** resulted in the extraction of several factors in each of the ecotypes separately, considering the three DTs. Since only one factor was identified in the Ardabil, Kerman, Mashhad, and Parsabad ecotypes, the extraction method was considered based on the “simple” method. In the Bushehr and Isfahan ecotypes, two factors were identified, so the extraction was done using the “rotated” method. Consequently, in the Ardabil, Bushehr, Isfahan, Kerman, Mashhad, and Parsabad ecotypes, four, nine, 10, seven, seven, and 12 effective compounds were identified in the extraction of factors, respectively. So that among them, compounds with loading values ≥ 0.6 indicated the most prominent contribution in the extraction of each factor (**Supplementary Table S8**).

**Figure 10:**
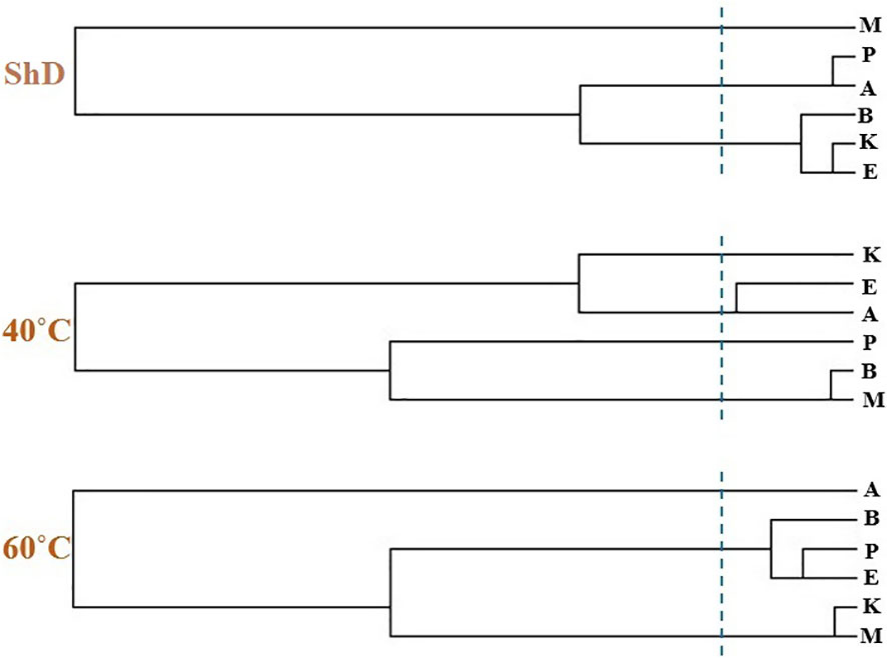
Cluster dendrogram of dill (*Anethum graveolens* L.) ecotypes separately in shade drying (ShD), 40°C, and 60°C drying temperatures (cluster cut line at 5 CASE Label Num) (A, Ardabil; M, Mashhad; E, Esfahan; P, Parsabad; B, Bushehr; K, Kerman ecotypes).

Ecotype grouping in cluster dendrograms based on the identified EO compounds at different DTs classified ecotypes into different subgroups (**Figure 10**). The discriminant function analysis test was used for the accuracy of grouping and determination of the cut line in the cluster dendrograms, which confirmed 100% accuracy (**Supplementary Table S9**). The classification of ecotypes according to the identified compounds under ShD led to the generation of three categories, including first (Isfahan, Kerman, and Bushehr ecotypes), second (Ardabil and Parsabad ecotypes), and third (Mashhad ecotype) subgroups. To identify the overlap of similar/dissimilar compounds produced at ShD, the Venn diagram was used as an alternative method to confirm the grouping of ecotypes in the cluster dendrogram.

**Figure 11:**
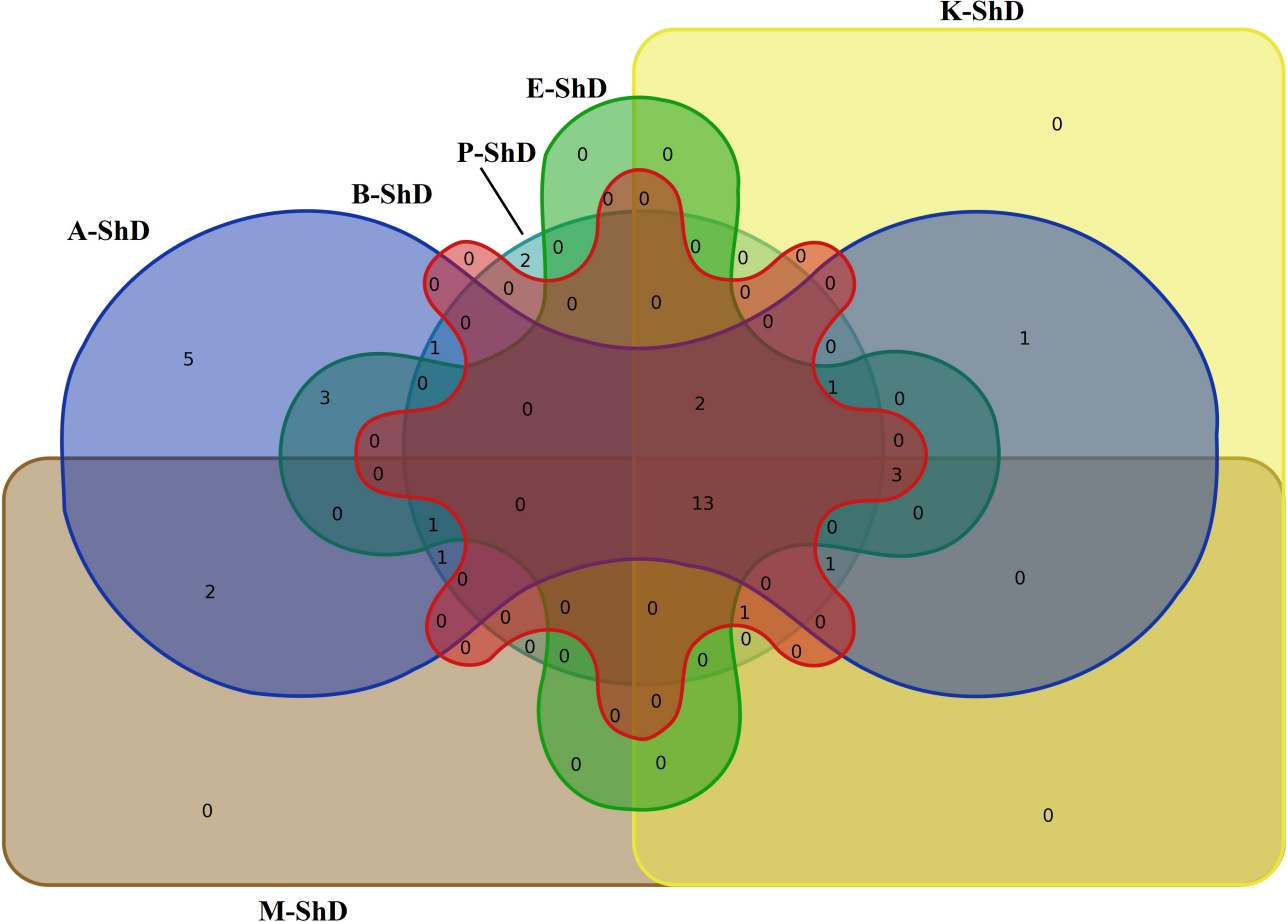
Venn plot for the number of increased/decreased similar/dissimilar identified essential oil compounds in dill (*Anethum graveolens* L.) ecotypes under shade drying (ShD) (A, Ardabil; M, Mashhad; E, Esfahan; P, Parsabad; B, Bushehr; K, Kerman ecotypes).

**Figure 12:**
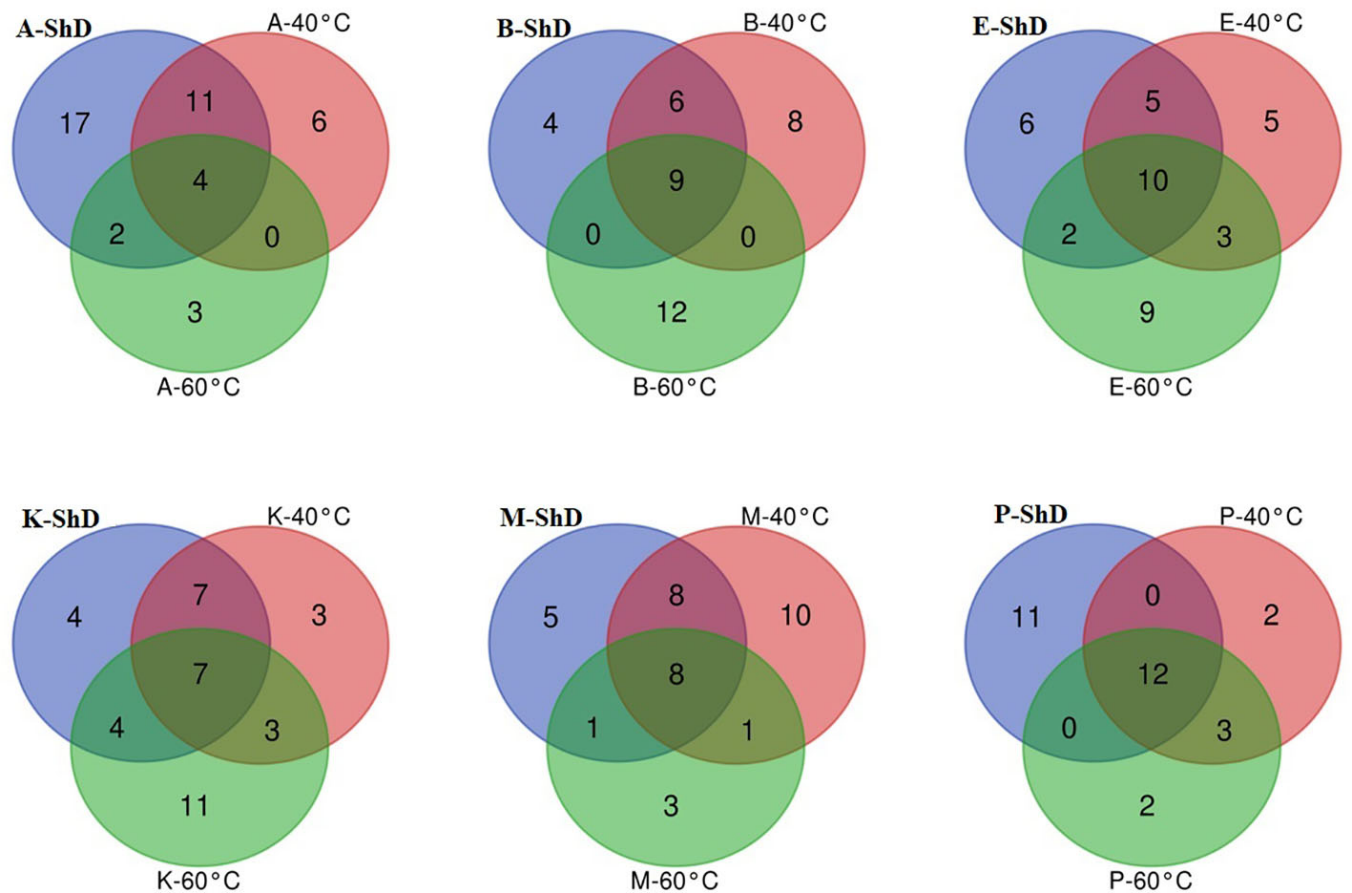
Venn plots for the number of increased/decreased similar/dissimilar identified essential oil compounds in dill (*Anethum graveolens* L.) ecotypes under three drying temperatures (shade drying (ShD), 40°C, and 60°C) as temperature raised (A, Ardabil; M, Mashhad; E, Esfahan; P, Parsabad; B, Bushehr; K, Kerman ecotypes).

As schematized in **Figure 11**, the Isfahan, Kerman, and Bushehr ecotypes have a high overlap in terms of the quality of the compounds produced. On the other hand, at ShD, the Ardabil and Parsabad ecotypes produced certain compounds with similar quality. At 40°C, the Mashhad, Bushehr, and Parsabad ecotypes were classified in the first subgroup, while the second subgroup included the Ardabil, Isfahan, and Kerman ecotypes (**Figure 10**). Alternatively, the Venn diagram confirmed the results of grouping at 40°C (**Supplementary Figure S1**). The grouping of ecotypes based on the results at 60°C DT divided the ecotypes into a first (Mashhad and Kerman ecotypes), a second (Isfahan, Parsabad, and Bushehr ecotypes), and a third subgroup (Ardabil ecotype) (**Figure 10**; **Supplementary Figure S2**). When examining the overlap of compounds produced in all DTs for each of the ecotypes separately, four, nine, 10, seven, eight, and 12 compounds were produced in the Ardabil, Bushehr, Isfahan, Kerman, Mashhad, and Parsabad ecotypes, respectively. On the other hand, 17 compounds at ShD, six compounds at 40°C, and three compounds at 60°C in the Ardabil ecotype; four, eight, and 12 compounds in the Bushehr ecotype; six, five, and nine compounds in the Isfahan ecotype; four, three, and 11 compounds in the Kerman ecotype; five, 10, and three compounds in the Mashhad ecotype; and 11, two, and two compounds in the Parsabad ecotype were produced at ShD, 40°C, and 60°C DT, respectively. This means that these compounds were not produced in the same ecotype at other DTs (**Figure 12**). Taken as a whole, in terms of the number of different produced compounds, the order was Ardabil–ShD (17 compounds) > Bushehr–60°C (12 compounds) > Parsabad–ShD = Kerman–60°C (11 compounds), and > Mashhad–40°C (10 compounds). Considering the many outstanding studies dealing with plant EOs, phytochemical diversity in some Apiaceae plants shows the influence of genetic background on EO yield and content in plant germplasm and ecotypes. In this context, the phytochemical diversity of *Bunium persicum* germplasm led to the classification of 15 ecotypes into five groups. Moreover, PCA decreased 16 variables to two PCs (PC1 and PC2) with 95.83% of the total variance. Also, the results of factor analysis indicated that the first four factors played the main role in explaining the total variance (Azimzadeh, 2012). Furthermore, Gholizadeh et al. (2021) in phytochemical diversity of *Anethum graveolens* L. ecotypes reported that the 30 ecotypes were classified into four different groups by cluster analysis, indicating high diversity of valuable ecotypes.

## Conclusion

The results of the current study apparently provide new information on the effects of drying temperatures × genetic backgrounds on EO yields, composition, and aroma profile of dill ecotypes. At three drying temperatures (ShD, 40°C, and 60°C) and six dill ecotypes (Ardabil, Mashhad, Esfahan, Parsabad, Bushehr, and Kerman ecotypes), the highest (1.86%) and lowest (0.04%) EO yields were related to the Parsabad ecotype at 40°C and Kerman ecotype at 60°C, respectively. Regardless, more compounds were produced at ShD than other DTs. However, the quantity and quality of the compound(s) need to be studied in detail in all three temperatures to determine which temperature is best for a particular compound. In terms of genetic background, the Parsabad ecotype (with 12 similar compounds) and the Esfahan ecotype (with 10 similar compounds) were each the most suitable ecotypes in terms of compositional quality at all three temperatures. However, the selection of an ecotype and the optimum temperature for a particular composition(s) depends on the objectives of the researchers and the requirements of the industry.

## Data availability statement

The original contributions presented in the study are included in the article/**Supplementary Material**, further inquiries can be directed to the corresponding author/s.

## Author contributions

KFK: conceptualization, methodology, investigation, data curation, formal analysis, software, and writing—original draft preparation. MM: supervision, funding acquisition, methodology, visualization, data curation, formal analysis, software, and editing. RF: software and writing—original draft preparation. ABK: validation, reviewing, and editing. SDD: investigation and drawing diagrams. FS: investigation. AMM: investigation.
