# Supplementary material for: Drying temperatures affect the qualitative–quantitative variation of aromatic profiling in Anethum graveolens L. ecotypes as an industrial–medicinal–vegetable plant
Source: Front Plant Sci. 2023 May 12;14:1137840. doi: 10.3389/fpls.2023.1137840 (PMC10214840; doi:10.3389/fpls.2023.1137840)
Supplement: Supplementary file 1 [file DataSheet_1.docx]

Supplementary Material

**Drying temperatures affect the qualitative-quantitative variation of aromatic profiling in *Anethum graveolens* L. ecotypes as an industrial-medicinal-vegetable plant**

| **Supplementary Table S1.**  Comparison of essential oil yield of dill (*Anethum graveolens* L.) in different plant organs. | | |
| --- | --- | --- |
| Reference | EO yield (%) | Plant organ |
| **This study** | **0.04-1.86 %** | **Aerial parts** |
| Orhan et al., 2013 | 0.07 and 0.23 % | Aerial parts |
| Santos et al., 2002 | 0.3 % | Aerial parts |
| Abdelkader and Lockwood, 2016 | 0.4 % | Aerial parts |
| Rostaei et al., 2018 | 1.09-1.84 g m^-2^ | Aerial parts |
| Rostaei et al., 2018 | ^˷^ 1.2-2.6 % | Aerial parts |
| Weisany, 2018 | 0.0251-0.683 % | Aerial parts |
| Amiri et al., 2015 | 0.51 % | Aerial parts |
| Rana and Blazquez, 2014 | 0.3 % | Aerial parts |
| Pino et al., 1995a | 0.3 % | Aerial parts |
| Vera and Chane-Ming, 1998 | 0.8 % | Herb |
| Said Al Ahl et al., 2015 | 0.08 % | Herb |
| Charles et al., 1995 | 0.10-0.30 % | Herb |
| Chubey and Dorrell, 1976 | 0.62 % | Herb |
| Vokk et al., 2011 | 0.56 and 0.65 % | Herb |
| Wall and Friesen, 1986 | 0.09-0.6 % | Herb |
| Dimov et al., 2019 | 0.90 % | Herb |
| Jafari and Hadavi, 2012 | 54.3 kg/ha | Herb |
| Gholinezhad, 2017 | 2.18-3.71 % | Herb |
| Ozliman et al., 2021 | 0.26-0.72 % | Herb |
| Nasiroleslami and Safaridolatabad, 2014 | 0.51-0.58 % | Herb |
| Gholizadeh et al., 2021 | 0.06-0.46 % | Herb |
| Jaafari et al., 2015 | 23.8-54.4 kg/ha | Herb |
| Popović et al., 2019 | 1.84 % | Herb |
| Salman et al., 2019 | 0.994 % | Herb |
| Safikhani Nasimi et al., 2011 | 0.8 % | Herb |
| Hao et al., 2021 | ^˷^ 0.1-3.9 % | Herb |
| Clark and Menary, 1984 | 43-104 kg/ha | Irrigated herb |
| Clark and Menary, 1984 | 41-77 kg/ha | Non-irrigated herb |
| Ghassemi-Golezani et al., 2011 | 0.35 % | Leaves |
| Radulescu et al., 2010 | 12 mL/kg | Leaves |
| Andalibi et al., 2011 | 0.216 g m^-2^ | Leaves |
| Ozliman et al., 2021 | 0.27-1.7 % | Leaves |
| Dimov et al., 2018 | 0.9 % | Leaves |
| Singh et al., 2017 | 0.9 % | Leaves |
| Ghassemi-Golezani and Solhi-Khajemarjan, 2021 | 7.33-22.53 g m^-2^ | Leaves and stems |
| Said Al Ahl et al., 2015 | 1.10 % | Vegetative stage |
| Jianu et al., 2012 | 0.67 % | Inflorescences |
| Dimov et al., 2019 | 0.36 % | Flowers |
| Ghassemi-Golezani et al., 2011 | 0.4 % | Flowers |
| Radulescu et al., 2010 | 32 ml/kg | Flowers |
| Dimov et al., 2018 | 0.36 % | Flowers |
| Sefidkon, 2001 | 0.21 % | Flowering shoot |
| Andalibi et al., 2011 | 6.146 g m^-2^ |  |
| Yazdani et al., 2004 | 1.1 and 3.38 % | Flowering and the beginning of seed formation |
| Hornok, 1980 | 3.26-3.95 % | Seeds |
| Charles et al., 1995 | 1.75-4 % | Seeds |
| Li et al., 2021 | 6.7 % | Seeds |
| Zeng et al., 2011 | 3.5 % | Seeds |
| Ghassemi-Golezani and Solhi-Khajemarjan, 2021 | 21.10-40.80 g m^-2^ | Seeds |
| Hassan and Elhassan, 2017 | 1.8 % | Seeds |
| Stanojević et al., 2015 | 2.12-2.80 % | Seeds |
| Darzi et al., 2012 | 2-2.21 % | Seeds |
| Ghassemi-Golezani et al., 2011 | 0.8 % | Seeds |
| Said Al Ahl et al., 2015 | 3.20 % | Seeds |
| Weisany et al., 2016 | ^˷^ 0.8-2.6 % | Seeds |
| Clark and Menary, 1984 | 57-73 kg/ha | Seeds |
| Sefidkon, 2001 | 2.42 % | Seeds |
| Singh et al., 2005 | 2.6 % | Seeds |
| Singh et al., 2017 | 2.4 % | Seeds |
| Chen et al., 2013 | 3.5 % | Seeds |
| Dobreva and Dimov, 2021 | 7.78-9.99 % | Seeds |
| Chen et al., 2014 | 3.5 % | Seeds |
| Sintim et al., 2015 | 1.14-1.31 % | Seeds |
| Bowes et al., 2004 | 0.68 to 3.95 % | Seeds |
| Khaldi et al., 2015 | 2.10 % | Seeds |
| Attique Babri et al., 2012 | 1.45 % | Seeds |
| Vokk et al., 2011 | 3.5 % | Seeds |
| Tian et al., 2011 | 3.5 % | Seeds |
| Tian et al., 2012 | 3.5 % | Seeds |
| Yili et al., 2009 | 4.2 % | Seeds |
| Bailer et al., 2001 | 2.6-4.6 % | Seeds |
| Shahmohammadi et al., 2014 | 39.7 kg/ha | Seeds |
| Ozliman et al., 2021 | 4.58-6.18 % | Seeds |
| Embong et al., 1977 | 1.2-1.8 % | Seeds |
| Dimov et al., 2017 | 0.9472* | Seeds |
| Jianu et al., 2012 | 2.91 % | Mature seeds |
| Jianu et al., 2012 | 0.92 % | Immature seeds |
| Jirovetz et al., 2003 | 3.8 % | Original seeds |
| Jirovetz et al., 2003 | 4 % | Stored seeds |
| Yazdani et al., 2004 | 3.3 and 3.4 % | Seeds in the green stage |
| Yazdani et al., 2004 | 2.4 and 2.49 % | Ripped and brown seeds |
| Kapoor et al., 2002 | 2.2 to 4 % | Fruits |
| Callan et al., 2007 | 44.6-56 kg/ha | Fruits |
| Radulescu et al., 2010 | 34 mL/kg | Fruits |
| Ruangamnart et al., 2015 | 1.05 and 2.01 % | Fruits |
| Santos et al., 2002 | 2 % | Fruits |
| Dimov et al., 2019 | 3.61 % | Fruits |
| Abdelkader and Lockwood, 2016 | 2.2 % | Fruits |
| Dimov et al., 2018 | 3.61 % | Fruits |
| Jianu et al., 2012 | 0.11 % | Stems |
| Madandoust and Fooladchang, 2018 | 2-2.25 % | Stems and leaves |
| Santos et al., 2002 | 0.06 % | Roots |
| Abdelkader and Lockwood, 2016 | 0.07 % | Parent roots |
| Santos et al., 2002 | 0.02 % | Hairy roots |
| Abdelkader and Lockwood, 2016 | 0.1 % | Photoperiod grown hairy roots |
| Abdelkader and Lockwood, 2016 | 0.08 % | Dark-grown hairy roots |
| Abdelkader and Lockwood, 2016 | 0.2 % | Shoot culture |
| * Specific gravity ( $d_{20}^{20}$) | | |

| **Supplementary Table S2**  Comparison of main EO compounds from aerial parts of dill (*Anethum graveolens* L.) plants in this and previous studies (%). | | | | | | | |
| --- | --- | --- | --- | --- | --- | --- | --- |
| Compound  Reference | α-Thujene | α-Phellandrene | *p*-Cymene | β-Phellandrene | Dill ether | Germacrene D | Dill apiole |
| **This study** | **0.09-0.73** | **2.17-57.49** | **5.08-9.87** | **0.07-14.79** | **0.37-8.84** | **0.13-10.91** | **2.29-35.3** |
| (Pino et al., 1995a) | 1.06 | 24.88 | 14.38 | 8.21 | - | - | 0.31 |
| (Santos et al., 2002) | - | 62.1 | - | 7.3 | - | - | 9.7 |
| (Orhan et al., 2013) | 0.211 and 0.287 | 27.940 and 47.748 | 10.247 | 7.916 | 9.841 and 17.344 | 0.181 and 0.373 | 1.224 and 3.797 |
| (Rana and Blazquez, 2014) | - | 31.8 | 5.3 | - | 13.2 | - | - |
| (Abdelkader and Lockwood, 2016) | - | 0.3 | 20.5 | 4.6 | 38.5 | - | 3.5 |
| (Amiri et al., 2015) | - | - | - | 19.89-27.56 | 2.85-15.27 | - | 0.36-15.27 |
| (Amanpour et al., 2017) | - | 160 * | - | - | 7.8 * | - | 6.66 * |
| (Rostaei et al., 2018) | 0.01-0.05 | 10.79-34.49 | 21.66-33.66 | - | 1.47-5.88 | 0.03-0.18 | 1.35-12.14 |
| (Weisany, 2018) | - | 2.14 and 2.45 | - | 0.39 and 0.47 | 0.6-0.7 | - | 48.57 and  49.75 |
| *= These numbers are in μg/g. | | | | | | | |

| **Supplementary Table S3**  Analysis of variance (mean squares) for three similar volatile compounds evaluated at three drying temperatures (shade drying, 40 °C, and 60 °C) and six dill (*Anethum graveolens* L.) ecotypes. | | | | |
| --- | --- | --- | --- | --- |
|  |  |  | Mean Square |  |
| S.O.V | DF | α-Phellandrene | Germacrene D | Dill apiole |
| Block (R) | 2 | 5881.306 ^***^ | 1.880 ^***^ | 1.150 ^***^ |
| Ecotype (A) | 5 | 106.606 ^ns^ | 0.718 ^**^ | 0.026 ^***^ |
| Drying Temperature (B) | 2 | 10.850 ^ns^ | 6.852E-5 ^ns^ | 8.760E-5 ^ns^ |
| A×B | 10 | 17.301 ^ns^ | 0.001 ^ns^ | 8.330E-5 ^ns^ |
| Error | 34 | 212.418 | 0.201 | 0.002 |
| Note: S.O.V= Source Of Variation, DF= Degree of Freedom, ^**^= Significant at the 0.01 probability level, ^ns^= not significant. | | | | |

| **Supplementary Table S4A**  Analysis of variance (mean squares) for essential oils compounds in dill (*Anethum graveolens* L.) ecotypes under shade drying. | | | | | | | | | |
| --- | --- | --- | --- | --- | --- | --- | --- | --- | --- |
| β-Phellandrene | α-Terpinene | α-Phellandrene | β-Myrcene | β-Pinene | Sabinene | Camphene | α-Thujene | DF | S.O.V |
| 0.008 ^ns^ | 0.000 ^ns^ | 2.490 ^ns^ | 0.001 ^**^ | 2.222E-5 ^ns^ | 3.889E-5 ^ns^ | 2.140E-5 ^ns^ | 0.000 ^**^ | 2 | Block |
| 1.454 ^***^ | 0.002 ^***^ | 65.905 ^ns^ | 0.001 ^***^ | 0.003 ^***^ | 0.000 ^***^ | 0.000 ^ns^ | 0.002 ^***^ | 5 | Treat |
| 0.004 | 5.667E-5 | 32.946 | 4.556E-5 | 8.889E-5 | 1.889E-5 | 4.708E-5 | 4.000E-5 | 10 | Error |
|  |  |  |  |  |  |  |  |  |  |
| **Supplementary Table S4A (Continued).** | | | | | | | | | |
|  |  |  | Neophytadiene | Dill apiole | Germacrene D | Carvacrol | Dill ether | DF | S.O.V |
|  |  |  | 7.222E-5 ^ns^ | 0.002 ^ns^ | 0.002 ^ns^ | 0.000 ^**^ | 0.001 ^ns^ | 2 | Block |
|  |  |  | 0.473 ^***^ | 1.383 ^***^ | 3.861 ^***^ | 0.007 ^***^ | 11.808 ^***^ | 5 | Treat |
|  |  |  | 1.889E-5 | 0.002 | 0.001 | 2.222E-5 | 0.007 | 10 | Error |
|  |  |  |  |  |  |  |  |  |  |
| **Supplementary Table S4B**  Analysis of variance (mean squares) for essential oils compounds in dill (*Anethum graveolens* L.) ecotypes under 40°C drying temperature. | | | | | | | | | |
| Dill apiole | Myristicin | Germacrene D | Dill ether | α-Terpinene | α**-**Phellandrene | β-Pinene | Sabinene | DF | S.O.V |
| 0.048 ^ns^ | 8.889E-5 ^ns^ | 0.003 ^ns^ | 0.010 ^ns^ | 3.889E-5 ^ns^ | 6.565 ^ns^ | 2.222E-5 ^ns^ | 0.000 ^ns^ | 2 | Block |
| 86.540 ^***^ | 1.181 ^***^ | 3.084 ^***^ | 17.799 ^***^ | 0.002 ^***^ | 50.523 ^***^ | 0.001 ^***^ | 0.003 ^***^ | 5 | Treat |
| 0.043 | 0.001 | 0.002 | 0.012 | 1.889E-5 | 3.961 | 8.889E-6 | 3.556E-5 | 10 | Error |
|  |  |  |  |  |  |  |  |  |  |
| **Supplementary Table S4C**  Analysis of variance (mean squares) for essential oils compounds in dill (*Anethum graveolens* L.) ecotypes under 60°C drying temperature. | | | | | | | | | |
|  |  |  |  |  | Dill apiole | Germacrene D | α-Phellandrene | DF | S.O.V |
|  |  |  |  |  | 1.638 ^ns^ | 0.683 ^**^ | 2.926 ^ns^ | 2 | Block |
|  |  |  |  |  | 241.670 ^***^ | 38.241 ^***^ | 511.159 ^***^ | 5 | Treat |
|  |  |  |  |  | 2.475 | 0.103 | 5.915 | 10 | Error |
| Note: S.O.V= Source Of Variation, DF= Degree of Freedom, *= Significant at the 0.05 probability, **= Significant at the 0.01 probability, levels ***= Significant at the 0.001 probability levels, ns= not significant. | | | | | | | | | |

| **Supplementary Table S5**  Mean comparison of significant essential oil compounds in dill (*Anethum graveolens* L.) ecotypes under shade drying. | | | | | | |
| --- | --- | --- | --- | --- | --- | --- |
|  | Ardabil | Mashhad | Esfahan | Parsabad | Bushehr | Kerman |
| α-Thujene | 0.56 ^c^ | 0.63 ^a^ | 0.56 ^c^ | 0.603 ^b^ | 0.56 ^c^ | 0.59 ^b^ |
| Sabinene | 0.21 ^c^ | 0.24 ^a^ | 0.21 ^c^ | 0.22 ^b^ | 0.213 ^c^ | 0.22 ^b^ |
| β-Pinene | 0.29 ^a^ | 0.223 ^c^ | 0.22 ^c^ | 0.25 ^b^ | 0.20 ^e^ | 0.213 ^d^ |
| β-Myrcene | 0.79 ^c^ | 0.84 ^a^ | 0.79 ^c^ | 0.813 ^b^ | 0.79 ^c^ | 0.81 ^b^ |
| α-Terpinene | 0.113 ^c^ | 0.13 ^b^ | 0.12 ^bc^ | 0.17 ^a^ | 0.173 ^a^ | 0.123 ^bc^ |
| β-Phellandrene | 12.95 ^e^ | 14.47 ^b^ | 14.07 ^c^ | 13.30 ^d^ | 14.79 ^a^ | 14.02 ^c^ |
| Dill ether | 3.73 ^f^ | 7.86 ^b^ | 5.78 ^d^ | 4.27 ^e^ | 8.84 ^a^ | 6.20 ^c^ |
| Carvacrol | 0.33 ^b^ | 0.303 ^c^ | 0.34 ^a^ | 0.20 ^e^ | 0.303 ^c^ | 0.29 ^d^ |
| Germacrene D | 3.38 ^a^ | 0.81 ^f^ | 1.77 ^c^ | 3.21 ^b^ | 0.92 ^e^ | 1.30 ^d^ |
| Dill apiole | 2.65 ^d^ | 3.19 ^c^ | 3.22 ^c^ | 2.29 ^e^ | 3.81 ^b^ | 4.09 ^a^ |
| Neophytadiene | 0.98 ^b^ | 0.193 ^e^ | 0.45 ^c^ | 1.03 ^a^ | 0.163 ^f^ | 0.24 ^d^ |
|  |  |  |  |  |  |  |
| **Supplementary Table S5 (Continued).** 40° C drying temperature. | | | | | | |
| Sabinene | 0.183 ^bc^ | 0.193 ^ab^ | 0.203 ^a^ | 0.120 ^e^ | 0.170 ^c^ | 0.153 ^d^ |
| β-Pinene | 0.10 ^b^ | 0.113 ^a^ | 0.11 ^a^ | 0.06 ^d^ | 0.11 ^a^ | 0.093 ^c^ |
| α-Phellandrene | 44.54 ^ab^ | 40.82 ^bc^ | 43.82 ^ab^ | 35.79 ^d^ | 39.46 ^c^ | 47.30 ^a^ |
| α-Terpinene | 0.153 ^a^ | 0.13 ^b^ | 0.10 ^c^ | 0.09 ^d^ | 0.08 ^e^ | 0.10 ^c^ |
| Dill ether | 8.03 ^a^ | 4.69 ^c^ | 2.79 ^d^ | 6.04 ^b^ | 1.70 ^f^ | 2.41 ^e^ |
| Germacrene D | 0.29 ^e^ | 0.83 ^d^ | 1.32 ^c^ | 0.133 ^f^ | 1.873 ^b^ | 2.803 ^a^ |
| Myristicin | 1.723 ^a^ | 1.233 ^c^ | 0.47 ^e^ | 1.653 ^b^ | 0.613 ^d^ | 0.27 ^f^ |
| Dill apiole | 10.84 ^e^ | 23.87 ^a^ | 12.91 ^d^ | 18.48 ^b^ | 23.68 ^a^ | 17.51 ^c^ |
|  |  |  |  |  |  |  |
| **Supplementary Table S5 (Continued).** 60° C drying temperature. | | | | | | |
| α-Phellandrene | 2.17 ^c^ | 16.43 ^b^ | 35.56 ^a^ | 32.09 ^a^ | 32.16 ^a^ | 15.71 ^b^ |
| Germacrene D | 10.91 ^a^ | 5.26 ^b^ | 4.96 ^b^ | 0.14 ^e^ | 2.94 ^d^ | 3.76 ^c^ |
| Dill apiole | 15.76 ^d^ | 35.30 ^a^ | 19.56 ^c^ | 23.06 ^c^ | 16.08 ^b^ | 35.14 ^a^ |
| Note: Means followed by the same letters in each column are not significantly different. | | | | | | |

| **Supplementary Table S6A**  Analysis of variance (mean squares) for essential oil compounds in Ardabil dill (*Anethum graveolens* L.) ecotype under three drying temperatures (shade drying, 40°C, and 60°C). | | | | | | | | | | | |
| --- | --- | --- | --- | --- | --- | --- | --- | --- | --- | --- | --- |
|  |  |  |  |  |  | Neophytadiene | Dill apiole | Germacrene D | α-Phellandrene | DF | S.O.V |
|  |  |  |  |  |  | 0.039 ^ns^ | 0.002 ^ns^ | 0.043 ^ns^ | 0.320 ^ns^ | 2 | Block |
|  |  |  |  |  |  | 73.594^***^ | 131.588^***^ | 89.562^***^ | 2218.025^***^ | 2 | Treat |
|  |  |  |  |  |  | 0.042 | 0.046 | 0.049 | 0.161 | 4 | Error |
|  |  |  |  |  |  |  |  |  |  |  |  |
| **Supplementary Table S6B.** Bushehr ecotype under three drying temperatures (shade drying, 40°C, and 60°C). | | | | | | | | | | | |
|  | Dill apiole | Germacrene D | Carvacrol | Dill ether | *p*-Cymene | α-Terpinene | α-Phellandrene | β-Myrcene | α-Thujene | DF | S.O.V |
|  | 0.441 ^ns^ | 0.126 ^ns^ | 0.001 ^ns^ | 0.004 ^ns^ | 0.082 ^ns^ | 0.001 ^ns^ | 2.236 ^ns^ | 0.001^*^ | 0.000 ^ns^ | 2 | Block |
|  | 301.473^***^ | 3.064 ^**^ | 0.016 ^**^ | 62.225 ^***^ | 10.663 ^**^ | 0.007 ^*^ | 407.712 ^***^ | 0.116 ^***^ | 0.091 ^***^ | 2 | Treat |
|  | 0.353 | 0.160 | 0.001 | 0.008 | 0.124 | 0.000 | 2.447 | 6.111E-5 | 6.111E-5 | 4 | Error |
|  |  |  |  |  |  |  |  |  |  |  |  |
| **Supplementary Table S6C.** Esfahan ecotype under three drying temperatures (shade drying, 40°C, and 60°C). | | | | | | | | | | | |
| Dill apiole | Germacrene D | Carvacrol | Dill ether | α-Terpinolene | β-Phellandrene | α-Terpinene | α-Phellandrene | β-Myrcene | α-Thujene | DF | S.O.V |
| 0.018 ^ns^ | 0.001^ns^ | 0.001^ns^ | 0.002 ^ns^ | 1.111E-5 ^ns^ | 0.024 ^ns^ | 7.778E-5 ^ns^ | 1.038 ^ns^ | 1.111E-5 ^ns^ | 4.444E-5 ^ns^ | 2 | Block |
| 202.634^***^ | 11.805^***^ | 0.032 ^*^ | 19.367^***^ | 0.007^***^ | 147.514^***^ | 0.001^**^ | 301.432^***^ | 0.202^***^ | 0.200^***^ | 2 | Treat |
| 0.026 | 0.003 | 0.002 | 0.004 | 6.111E-5 | 2.021 | 2.778E-5 | 0.483 | 0.000 | 6.111E-5 | 4 | Error |
|  |  |  |  |  |  |  |  |  |  |  |  |
| **Supplementary Table S6D.** Kerman ecotype under three drying temperatures (shade drying, 40°C, and 60°C). | | | | | | | | | | | |
|  |  |  | Hexahydrofarnesyl acetone | Dill apiole | Germacrene D | Carvacrol | 1-Terpineol | β-Phellandrene | α-Phellandrene | DF | S.O.V |
|  |  |  | 1.111E-5 ^ns^ | 4.444E-5 ^ns^ | 0.036 ^ns^ | 0.001 ^*^ | 3.333E-5 ^ns^ | 0.052 ^ns^ | 1.703 ^ns^ | 2 | Block |
|  |  |  | 0.067 ^***^ | 727.346 ^***^ | 4.625 ^***^ | 0.450 ^***^ | 0.003 ^**^ | 92.440 ^***^ | 1423.666 ^***^ | 2 | Treat |
|  |  |  | 1.111E-5 | 0.036 | 0.025 | 0.000 | 3.333E-5 | 0.092 | 1.441 | 4 | Error |
|  |  |  |  |  |  |  |  |  |  |  |  |
| **Supplementary Table S6E.** Mashhad ecotype under three drying temperatures (shade drying, 40°C, and 60°C). | | | | | | | | | | | |
|  |  |  | Palmitic acid | Neophytadiene | Dill apiole | Germacrene D | α-Phellandrene | β-Myrcene | α-Pinene | DF | S.O.V |
|  |  |  | 0.124 ^ns^ | 0.019 ^ns^ | 3.741 ^ns^ | 0.163 ^ns^ | 76.811 ^ns^ | 0.000 ^ns^ | 0.013 ^ns^ | 2 | Block |
|  |  |  | 18.664 ^***^ | 7.842 ^***^ | 794.680 ^***^ | 19.714 ^***^ | 696.199 ^**^ | 0.379 ^***^ | 5.648 ^***^ | 2 | Treat |
|  |  |  | 0.130 | 0.020 | 4.534 | 0.185 | 26.522 | 0.000 | 0.007 | 4 | Error |
|  |  |  |  |  |  |  |  |  |  |  |  |
| **Supplementary Table S6F.** Parsabad ecotype under three drying temperatures (shade drying, 40°C, and 60°C). | | | | | | | | | | | |
|  |  |  |  | α-Phellandrene | β-Myrcene | β-Pinene | Sabinene | Camphene | α-Thujene | DF | S.O.V |
|  |  |  |  | 14.432 ^ns^ | 0.001 ^**^ | 0.000 ^ns^ | 1.111E-5 ^ns^ | 0.000 ^ns^ | 0.000 ^*^ | 2 | Block |
|  |  |  |  | 326.144 ^**^ | 0.111 ^***^ | 0.004 ^***^ | 0.012 ^***^ | 0.001 ^**^ | 0.110 ^***^ | 2 | Treat |
|  |  |  |  | 21.955 | 2.778E-5 | 0.000 | 1.111E-5 | 4.444E-5 | 3.333E-5 | 4 | Error |
|  |  |  |  |  |  |  |  |  |  |  |  |
| **Supplementary Table S6F (Continued).** | | | | | | | | | | | |
|  |  |  |  | Dill apiole | Germacrene D | Dill ether | α-Terpinolene | γ-Terpinene | α-Terpinene | 2 | Block |
|  |  |  |  | 0.013 ^ns^ | 1.111E-5 ^ns^ | 0.025 ^ns^ | 4.444E-5 ^ns^ | 0.000 ^ns^ | 1.111E-5 ^ns^ | 2 | Treat |
|  |  |  |  | 357.366 ^***^ | 9.445 ^***^ | 2.572 ^***^ | 0.069 ^***^ | 0.001 ^*^ | 0.009 ^***^ | 4 | Error |
|  |  |  |  | 0.041 | 1.111E-5 | 0.014 | 1.111E-5 | 8.333E-5 | 0.000 | 2 | Block |
| Note: S.O.V= Source Of Variation, DF= Degree of Freedom, ^*^= Significant at the 0.05 probability, ^**^= Significant at the 0.01 probability, levels ^***^= Significant at the 0.001 probability levels, ^ns^= not significant. | | | | | | | | | | | |

| **Supplementary Table S7**  Rotated component matrix and total variance explained of essential oil compounds considering three drying temperatures separately in dill (*Anethum* *graveolens* L.) ecotypes. | | | | | | | | | | | | | | | | |
| --- | --- | --- | --- | --- | --- | --- | --- | --- | --- | --- | --- | --- | --- | --- | --- | --- |
| Compound Name | 25 °C | | | | | | 40 °C | | | | | 60 °C | | | |  |
|  | Component | | | | Communalities | | Component | | | Communalities | | Component | | Communalities | |  |
|  | 1 | 2 | 3 | 4 | Initial | Extraction | 1 | 2 | 3 | Initial | Extraction | 1 | 2 | Initial | Extraction |  |
| α-Thujene | -0.129 | **0.931** | 0.138 | -0.188 | 1.00 | 0.938 | **-** | **-** | **-** | - | - | - | - | - | - |  |
| Camphene | 0.289 | 0.159 | **0.929** | 0.103 | 1.00 | 0.983 | **-** | **-** | **-** | - | - | - | - | - | - |  |
| Sabinene | -0.291 | **0.930** | 0.128 | -0.043 | 1.00 | 0.968 | -0.005 | **0.946** | 0.162 | 1.00 | 0.921 | - | - | - | - |  |
| β-Pinene | **0.927** | 0.067 | 0.217 | 0.201 | 1.00 | 0.951 | -0.257 | **0.953** | -0.009 | 1.00 | 0.974 | - | - | - | - |  |
| β-Myrcene | -0.128 | **0.925** | 0.038 | -0.126 | 1.00 | 0.889 | **-** | **-** | **-** | - | - | - | - | - | - |  |
| α-Phellandrene | -0.048 | **-0.643** | -0.304 | 0.077 | 1.00 | 0.513 | -0.362 | 0.353 | **0.793** | 1.00 | 0.885 | **-0.917** | -0.325 | 1.00 | 0.946 |  |
| α-Terpinene | -0.178 | -0.061 | -0.096 | **-0.920** | 1.00 | 0.891 | **0.679** | 0.465 | 0.434 | 1.00 | 0.868 | - | - | - | - |  |
| β-Phellandrene | **-0.979** | 0.051 | -0.091 | -0.068 | 1.00 | 0.975 | **-** | **-** | **-** | - | - | - | - | - | - |  |
| Dill ether | **-0.927** | 0.132 | -0.147 | -0.068 | 1.00 | 0.903 | **0.940** | -0.142 | 0.278 | 1.00 | 0.981 | - | - | - | - |  |
| Carvacrol | -0.289 | -0.265 | -0.056 | **0.860** | 1.00 | 0.896 | **-** | **-** | **-** | - | - | - | - | - | - |  |
| Germacrene D | **0.955** | -0.186 | 0.181 | -0.129 | 1.00 | 0.996 | **-0.945** | 0.102 | 0.130 | 1.00 | 0.920 | **0.933** | -0.186 | 1.00 | 0.905 |  |
| Myristicin | **-** | **-** | **-** | **-** | - | - | **0.962** | -0.202 | -0.078 | 1.00 | 0.973 | **-** | **-** | - | - |  |
| Dill apiole | **-0.742** | -0.113 | -0.517 | 0.271 | 1.00 | 0.904 | -0.271 | 0.058 | **-0.864** | 1.00 | 0.824 | -0.075 | **0.987** | 1.00 | 0.979 |  |
| Neophytadiene | **0.938** | -0.117 | 0.243 | -0.209 | 1.00 | 0.996 | **-** | **-** | **-** | - | - | **-** | **-** | - | - |  |
| Eigenvalues | 6.236 | 4.018 | 2.332 | 2.000 | - | - | 3.434 | 2.218 | 1.694 | - | - | 2.507 | 1.246 | - | - |  |
| Variance (%) | 38.973 | 25.111 | 14.578 | 12.501 | - | - | 42.925 | 27.728 | 21.173 | - | - | 62.669 | 31.161 | - | - |  |
| Cumulative (%) | 38.973 | 64.084 | 78.662 | 91.163 | - | - | 42.925 | 70.653 | 91.826 | - | - | 62.669 | 93.830 | - | - |  |
| - Factor Loading values more than 0.6 were considered as significant (the bold numbers are the highest loading for each factors). - Extraction method is based on the Principal Component Analysis (PCA). | | | | | | | | | | | | | | | |  |

| **Supplementary Table S8**  Simple and rotated component matrix and total variance explained of essential oil compounds in dill (*Anethum graveolens* L.) ecotypes separately under drying temperatures | | | | | | | | | | | | | | | | | | | | |
| --- | --- | --- | --- | --- | --- | --- | --- | --- | --- | --- | --- | --- | --- | --- | --- | --- | --- | --- | --- | --- |
| Compound Name | Ardabil | | | Bushehr | | | | Esfahan | | | | Kerman | | | Mashhad | | | Parsabad | | |
|  | Simple | C | | Rotated | | C | | Rotated | | C | | Simple | C | | Simple | C | | Simple | C | |
|  | 1 | I | E | 1 | 2 | I | E | 1 | 2 | I | E | 1 | I | E | 1 | I | E | 1 | I | E |
| α-Thujene | - | - | - | **0.977** | 0.199 | 1.00 | 0.994 | **-0.994** | 0.107 | 1.00 | 0.999 | - | - | - | - | - | - | **0.996** | 1.00 | 0.992 |
| α-Pinene | - | - | - | - | - | - | - | - | - | - | - | - | - | - | **-0.992** | 1.00 | 0.985 | - | - | - |
| Camphene | - | - | - | - | - | - | - | - | - | - | - | - | - | - | - | - | - | **0.988** | 1.00 | 0.995 |
| Sabinene | - | - | - | - | - | - | - | - | - | - | - | - | - | - | - | - | - | **0.994** | 1.00 | 0.989 |
| β-Pinene | - | - | - | - | - | - | - | - | - | - | - | - | - | - | - | - | - | **0.999** | 1.00 | 0.997 |
| β-Myrcene | - | - | - | **0.928** | 0.364 | 1.00 | 0.994 | **-0.985** | 0.164 | 1.00 | 0.997 | - | - | - | **-0.997** | 1.00 | 0.993 | **0.988** | 1.00 | 0.976 |
| α-Phellandrene | **-0.988** | 1.00 | 0.975 | **0.974** | -0.224 | 1.00 | 1.00 | -0.490 | **0.867** | 1.00 | 0.992 | **-0.996** | 1.00 | 0.992 | **-0.940** | 1.00 | 0.883 | **0.938** | 1.00 | 0.880 |
| α-Terpinene | - | - | - | **0.708** | **-0.617** | 1.00 | 0.883 | -0.281 | **0.886** | 1.00 | 0.865 | - | - | - | - | - | - | **0.974** | 1.00 | 0.949 |
| *p*-Cymene | - | - | - | 0.396 | **0.909** | 1.00 | 0.984 | - | - | - | - | - | - | - | - | - | - | - | - | - |
| β-Phellandrene | - | - | - | - | - | - | - | 0.474 | **0.877** | 1.00 | 0.994 | **-0.996** | 1.00 | 0.992 | - | - | - | - | - | - |
| γ-Terpinene | - | - | - | - | - | - | - | - | - | - | - | - | - | - | - | - | - | **0.998** | 1.00 | 0.995 |
| α-Terpinolene | - | - | - | - | - | - | - | -0.330 | **0.935** | 1.00 | 0.983 | - | - | - | - | - | - | **0.999** | 1.00 | 0.997 |
| 1-Terpineol | - | - | - | - | - | - | - | - | - | - | - | **0.820** | 1.00 | 0.672 | - | - | - | - | - | - |
| Dill ether | - | - | - | **0.914** | -0.388 | 1.00 | 0.987 | -0.486 | **0.872** | 1.00 | 0.996 | - | - | - | - | - | - | **-0.639** | 1.00 | 0.408 |
| Carvacrol | - | - | - | 0.336 | **0.903** | 1.00 | 0.929 | **0.681** | **-0.608** | 1.00 | 0.833 | **0.991** | 1.00 | 0.983 | - | - | - | - | - | - |
| Germacrene D | **0.942** | 1.00 | 0.888 | **-0.971** | -0.011 | 1.00 | 0.943 | **0.949** | -0.315 | 1.00 | 0.999 | **0.917** | 1.00 | 0.842 | **0.968** | 1.00 | 0.936 | **0.997** | 1.00 | 0.995 |
| Dill apiole | **0.809** | 1.00 | .654 | -0.596 | **0.803** | 1.00 | 1.00 | 0.485 | **-0.872** | 1.00 | 0.995 | **0.982** | 1.00 | 0.964 | **0.852** | 1.00 | 0.726 | **-0.985** | 1.00 | 0.971 |
| Neophytadiene | **0.988** | 1.00 | 0.976 | - | - | - | - | - | - | - | - | - | - | - | **0.986** | 1.00 | 0.972 | - | - | - |
| Hexahydrofarnesyl acetone | - | - | - | - | - | - | - | - | - | - | - | **0.973** | 1.00 | 0.947 | - | - | - | - | - | - |
| Palmitic acid | - | - | - | - | - | - | - | - | - | - | - | - | - | - | **0.983** | 1.00 | 0.967 | - | - | - |
| Eigenvalues | 4.366 | - | - | 7.180 | 4.405 | - | - | 6.799 | 5.795 | - | - | 7.221 | - | - | 7.433 | - | - | 11.146 | - | - |
| Variance (%) | 87.318 | - | - | 59.834 | 36.712 | - | - | 52.299 | 44.580 | - | - | 90.259 | - | - | 92.915 | - | - | 92.885 | - | - |
| Cumulative (%) | 87.318 | - | - | 59.834 | 96.547 | - | - | 52.299 | 96.879 | - | - | 90.259 | - | - | 92.915 | - | - | 92.885 | - | - |
| - Factor Loading values more than 0.6 were considered as significant (the bold numbers are the highest loading for each factors). - Extraction method is based on the Principal Component Analysis (PCA). - C= Communalities; I= Initial; E= Extraction. | | | | | | | | | | | | | | | | | | | | |

| **Supplementary Table S9**  Discrimination function analysis for evaluating the accuracy of grouping of dill (*Anethum graveolens* L.) ecotypes under three drying temperatures (shade drying, 40°C, and 60°C) in cluster dendrogram. | | | | | | | | | | | | | |
| --- | --- | --- | --- | --- | --- | --- | --- | --- | --- | --- | --- | --- | --- |
|  | Grouping of clusters dendrogram | Shade drying | | | | 40 °C | | | | 60 °C | | | |
|  |  | 1 | 2 | 3 | Total | 1 | 2 | 3 | Total | 1 | 2 | 3 | Total |
|  | 1 | 3 | 0 | 0 | 3 | 2 | 0 | 0 | 2 | 2 | 0 | 0 | 2 |
| Number | 2 | 0 | 2 | 0 | 2 | 0 | 1 | 0 | 1 | 0 | 3 | 0 | 3 |
|  | 3 | 0 | 0 | 1 | 1 | 0 | 0 | 3 | 3 | 0 | 0 | 1 | 1 |
|  |  |  |  |  |  |  |  |  |  |  |  |  |  |
|  | 1 | 100 | 0 | 0 | 100 | 100 | 0 | 0 | 100 | 100 | 0 | 0 | 100 |
| Success rate (%) | 2 | 0 | 100 | 0 | 100 | 0 | 100 | 0 | 100 | 0 | 100 | 0 | 100 |
|  | 3 | 0 | 0 | 100 | 100 | 0 | 0 | 100 | 100 | 0 | 0 | 100 | 100 |
| Mean of success (%) | | **100** | | | | **100** | | | | **100** | | | |


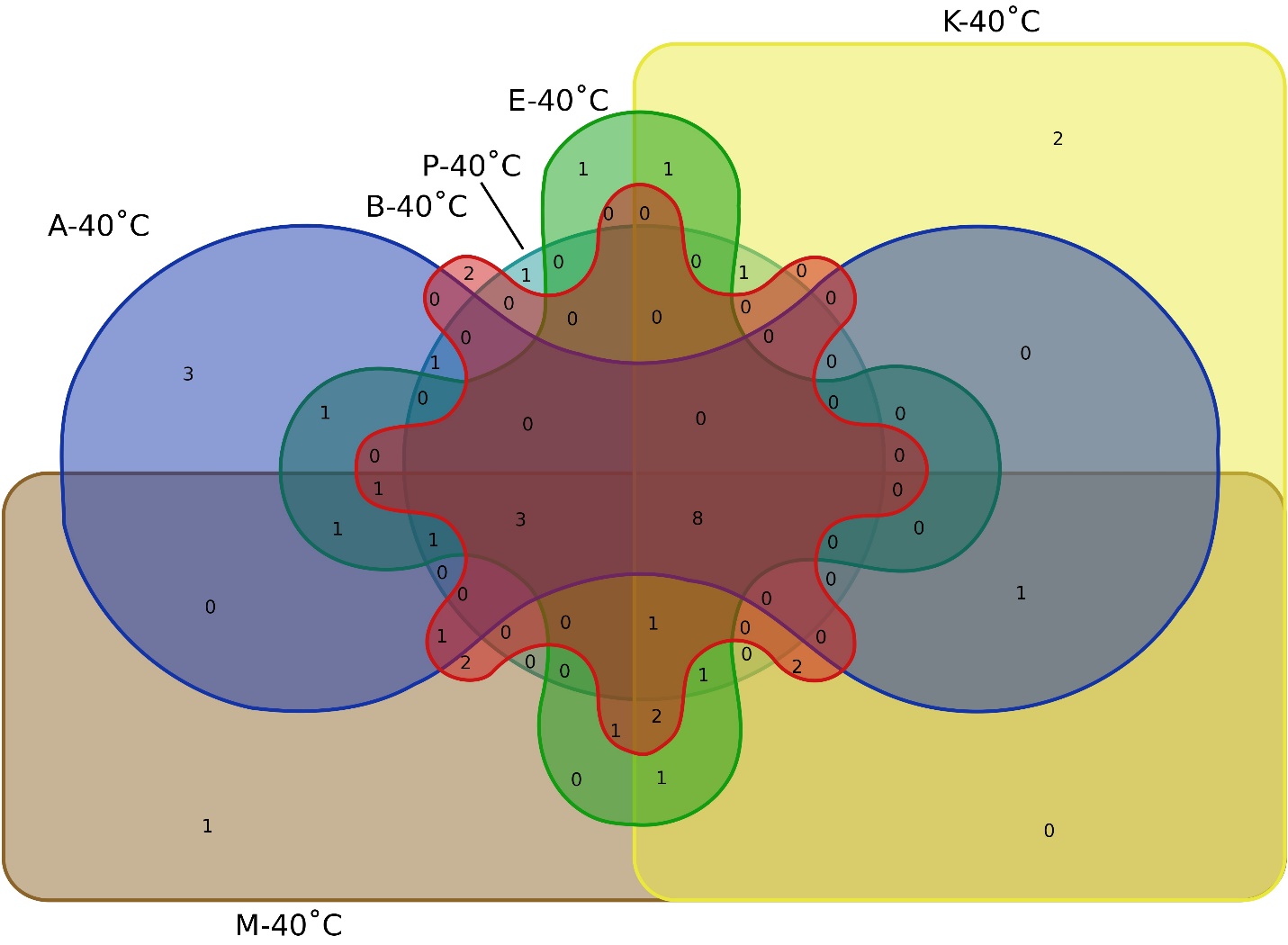


**Supplementary Figure S1.** Venn plot for the number of increased/decreased similar/dissimilar identified essential oil compounds in dill (*Anethum graveolens* L.) ecotypes under 40 °C drying temperature (A: Ardabil, M: Mashhad; E: Esfahan; P: Parsabad; B: Bushehr; K: Kerman ecotypes).

**
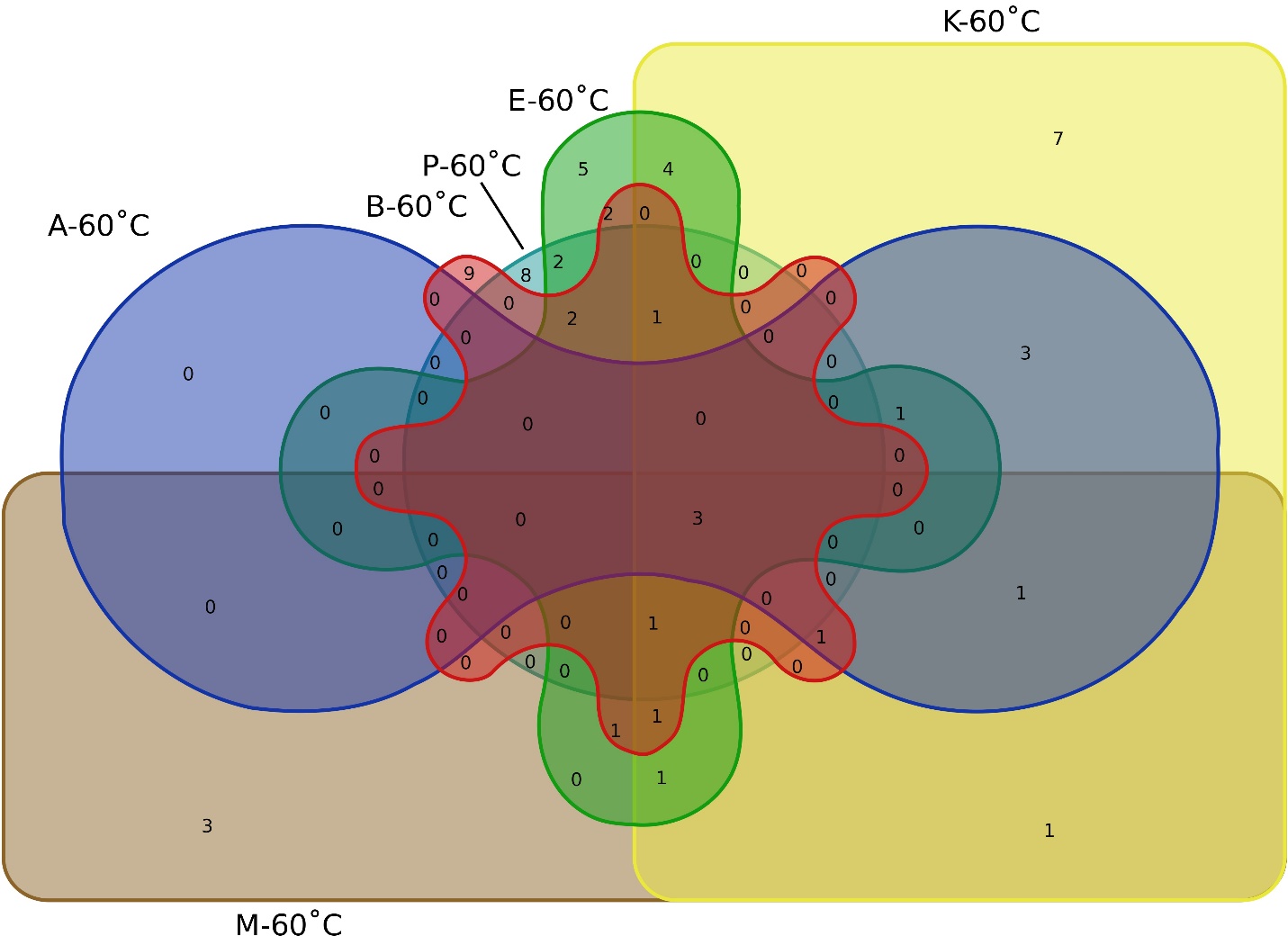
**

**Supplementary Figure S2.** Venn plot for the number of increased/decreased similar/dissimilar identified essential oil compounds in dill (*Anethum graveolens* L.) ecotypes under 60 °C drying temperature (A: Ardabil, M: Mashhad; E: Esfahan; P: Parsabad; B: Bushehr; K: Kerman ecotypes).

**References:**

Abdelkader, M.S.A., Lockwood, G.B. (2016). Essential oils from the plant, hairy root cultures and shoot cultures of Egyptian *Anethum graveolens* (dill). *J. Essent. Oil Res.* 28, 104–112. https://doi.org/10.1080/10412905.2015.1091790

Amanpour, A., Kelebek, H., Selli, S. (2017). Aroma constituents of shade-dried aerial parts of Iranian dill (*Anethum graveolens* L.) and savory (*Satureja sahendica* Bornm.) by solvent-assisted flavor evaporation technique. *J. Food Meas. Charact.* 11, 1430–1439. https://doi.org/10.1007/s11694-017-9522-5

Amiri, H., Emami, H., Abdollahi, S.F. (2015). Effects of drougth stress on the essential oil content and composition of dill (*Anethum graveolens* L.). *Plant Process Funct.* 3, 143–150. https://doi.org/20.1001.1.23222727.1393.3.10.4.3

Andalibi, B., Zehtab Salmasi, S., Ghassemi Gholezani, K., Saba, J. (2011). Changes in essential oil yield and composition at different parts of dill (*Anethum graveolens* L.) under limited irrigation conditions. *J. Agric. Sci. Sustain. Prod.* 21, 11–24.

Attique Babri, R., Khokhar, I., Mahmood, Z., Mahmud, S. (2012). Chemical composition and insecticidal activity of the essential oil of *Anethum graveolens* L. *Sci.Int.*(Lahore) 24, 453–455.

Bailer, J., Aichinger, T., Hackl, G., de Hueber, K., Dachler, M. (2001). Essential oil content and composition in commercially available dill cultivars in comparison to caraway. *Ind. Crops Prod.* 14, 229–239. https://doi.org/10.1016/S0926-6690(01)00088-7

Bowes, K.M., Zheljazkov, V.D., Caldwell, C.D., Pincock, J.A., Roberts, J.C. (2004). Influence of seeding date and harvest stage on yields and essential oil composition of three cultivars of dill (*Anethum graveolens* L.) grown in Nova Scotia. *Can. J. Plant Sci.* 84, 1155–1160. https://doi.org/10.4141/P03-202

Callan, N.W., Johnson, D.L., Westcott, M.P., Welty, L.E. (2007). Herb and oil composition of dill (*Anethum graveolens* L.): Effects of crop maturity and plant density. *Ind. Crops Prod.* 25, 282–287. https://doi.org/10.1016/j.indcrop.2006.12.007

Charles, D.J., Simon, J.E., Widrlechner, M.P. (1995). Characterization of essential oil of dill (*Anethum graveolens* L.). *J. Essent. Oil Res.* 7, 11–20. https://doi.org/10.1080/10412905.1995.9698456

Chen, Y., Zeng, H., Tian, J., Ban, X., Ma, B., Wang, Y. (2013). Antifungal mechanism of essential oil from *Anethum graveolens* seeds against *Candida albicans*. *J. Med. Microbiol.* 62, 1175–1183. https://doi.org/10.1099/jmm.0.055467-0

Chen, Y., Zeng, H., Tian, J., Ban, X., Ma, B., Wang, Y. (2014). Dill (*Anethum graveolens* L.) seed essential oil induces *Candida albicans* apoptosis in a metacaspase-dependent manner. *Fungal Biol.* 118, 394–401. https://doi.org/10.1016/j.funbio.2014.02.004

Chubey, B.B., Dorrell, D.G. (1976). Changes in the chemical composition of dill oil during hydrodistillation. *Can. J. Plant Sci.* 56, 619–622. https://doi.org/10.4141/cjps76-098

Clark, R.J., Menary, R.C. (1984). The effect of harvest date on the yield and composition of Tasmanian dill oil (*Anethum graveolens* L.). *J. Sci. Food Agric.* 35, 1186–1190. https://doi.org/10.1002/jsfa.2740351108

Darzi, M.T., Hadi, M.H.S., Rejali, F. (2012). Effects of the application of vermicompost and nitrogen fixing bacteria on quantity and quality of the essential oil in dill (*Anethum graveolens*). *J. Med. Plants Res.* 6, 3793–3799. https://doi.org/10.5897/jmpr12.370

Dimov, M., Dobreva, K., Damyanova, S., Stoyanova, A. (2017). Chemical composition, antioxidant and antimicrobial activities of dill essential oils (*Anethum graveolens* L.). Annu. *Assen Zlatarov Univ. Burgas* XLVI, 37–42.

Dimov, M., Georgieva, K., Denev, Y., Dobreva, K., Stoyanova, A. (2018). Analysis of the chemical composition of dill essential oils (*Anethum graveolens* L .) by the method of infra-red spectroscopy. *НАУЧНИ ТРУДОВЕ НА УНИВЕРСИТЕТ ПО ХРАНИТЕЛНИ ТЕХНОЛОГИИ - ПЛОВДИВ* 65, 55–60.

Dimov, M.D., Dobreva, K.Z., Stoyanova, A.S. (2019). Chemical composition of the dill essential oils (*Anethum graveolens* L.) from Bulgaria. *Bulg. Chem. Commun.* 51, 214–216.

Dobreva, K.Z., Dimov, M.D. (2021). Study of the changes in the chemical composition of Bulgarian dill essential oils. *IOP Conf. Ser. Mater. Sci.* Eng. 1031. https://doi.org/10.1088/1757-899X/1031/1/012108

Embong, M.B., Hadziyev, D., Molnar, S. (1977). Essential oils from spices grown in Alberta dill seed oil, *Anethum graveolens*, L. (Umbelliferae). *Can. Inst. Food Sci. Technol. J.* 10, 208–214. https://doi.org/10.1016/s0315-5463(77)73504-7

Ghassemi-Golezani, K., Solhi-Khajemarjan, R. (2021). Changes in growth and essential oil content of dill (*Anethum graveolens*) organs under drought stress in response to salicylic acid. *J. Plant Physiol. Breed*. 11, 33–47. https://doi.org/10.22034/JPPB.2021.13717

Ghassemi-Golezani, K., Zehtab-Salmasi, S., Dastborhan, S. (2011). Changes in essential oil content of dill (*Anethum graveolens*) organs under salinity stress. *J. Med. Plants Res.* 5, 3142–3145.

Gholinezhad, E. (2017). Effect of drought stress and fe nano-fertilizer on seed yield, morphological traits, essential oil percentage and yield of dill (*Anethum graveolens* L.). *J. Essent. Oil-Bearing Plants* 20, 1006–1017. https://doi.org/10.1080/0972060X.2017.1362999

Gholizadeh, A., Mohebodini, M., Ebadi, A., Chamani, E. (2021). Evaluation of genetic diversity of *Anethum graveolens* L. different ecotypes based on morphological traits and essential oil percentage of shoot. *Iran. J. Hortic. Sci.* 52, 581–592. https://doi.org/10.22059/ijhs.2020.294265.1751

Hao, Y., Kang, J., Guo, X., Yang, R., Chen, Y., Li, J., Shi, L. (2021). Comparison of nutritional compositions and essential oil profiles of different parts of a dill and two fennel cultivars. *Foods* 10, 1–13. https://doi.org/10.3390/foods10081784

Hassan, O.M., Elhassan, I.A. (2017). Characterization of essential oils from fruits of Umbelliferous crop cultivated in Sudan I. *Pimpinella anisum* L (Anise) and *Anethum graveolens* L. (Dill). *J. Pharmacogn. Phytochem.* 6, 109–112

Hornok, L. (1980). effect of nutrition supply on yield of dill (*Anethum graveolens* L.) and the essential oil content. *Acta Hortic.* 96, 337–342. https://doi.org/10.17660/ActaHortic.1980.96.36

Jaafari, N., Mirzaei, H.H., Hasanloo, T., Hadavi, E. (2015). Manipulating essential oil composition of dill (*Anethum graveolens* L.) by using preharvest foliar sprays of citric acid and malic acid. *J. Essent. Oil-Bearing Plants* 18, 556–560. https://doi.org/10.1080/0972060X.2014.977566

Jafari, N., Hadavi, E. (2012). Growth and essential oil yield of dill (*Anethum graveolens*) as affected by foliar sprays of citric acid and malic acid. *Acta Hortic.* 955, 287–290. https://doi.org/10.17660/ActaHortic.2012.955.42

Jianu, C., Misca, C., Georgeta, P.O.P., Rusu, L.C., Ardelean, L., Gruia, A.T. (2012). Chemical composition and antimicrobial activity of essential oils obtained from dill (*Anethum graveolens* L.) grown in Western Romania. *Rev. Chim.* 63, 641–645.

Jirovetz, L., Buchbauer, G., Stoyanova, A.S., Georgiev, E. V., Damianova, S.T. (2003). Composition, quality control, and antimicrobial activity of the essential oil of long-time stored dill (*Anethum graveolens* L.) seeds from Bulgaria. J. Agric. *Food Chem.* 51, 3854–3857. https://doi.org/10.1021/jf030004y

Kapoor, R., Giri, B., Mukerji, K.G. (2002). *Glomus macrocarpum*: A potential bioinoculant to improve essential oil quality and concentration in dill (*Anethum graveolens* L.) and carum (*Trachyspermum ammi* (Linn.) Sprague). *World J. Microbiol. Biotechnol.* 18, 459–463. https://doi.org/10.1023/A:1015522100497

Khaldi, Achraf, Meddah, B., Moussaoui, A., Sonnet, P. (2015). Chemical composition and antifungal activity of essential oil of *Anethum graveolens* L . from South-western Algeria ( Bechar ). *J. Chem. Phramaceutical Res.* 7, 615–620.

Li, H., Zhou, W., Hu, Y., Mo, H., Wang, J., Hu, L. (2021). GC-MS analysis of essential oil from *Anethum graveolens* L (dill) seeds extracted by supercritical carbon dioxide. *Trop. J. Pharm. Res.* 18, 1291–1296. https://doi.org/10.4314/TJPR.V18I6.21

Madandoust, M., Fooladchang, M. (2018). Effect of nitrogen fertilizer on essential oil content and its compositions in *Anethum graveolens* L. *J. Essent. Oil-Bearing Plants* 21, 1266–1271. https://doi.org/10.1080/0972060X.2018.1544934

Nasiroleslami, E., Safaridolatabad, S. (2014). The comparison of organic and biologic fertilizers effects on growth and essential oil of dill (*Anethum graveolens* L.). *Int. J. Biosci.* 5, 65–74. https://doi.org/10.12692/ijb/5.7.65-74

Orhan, I.E., Senol, F.S., Ozturk, N., Celik, S.A., Pulur, A., Kan, Y. (2013). Phytochemical contents and enzyme inhibitory and antioxidant properties of *Anethum graveolens* L. (dill) samples cultivated under organic and conventional agricultural conditions. *Food Chem. Toxicol.* 59, 96–103. https://doi.org/10.1016/j.fct.2013.05.053

Ozliman, S., Yaldiz, G., Camlica, M., Ozsoy, N. (2021). Chemical components of essential oils and biological activities of the aqueous extract of *Anethum graveolens* L. grown under inorganic and organic conditions. *Chem. Biol. Technol. Agric.* 8, 1–16. https://doi.org/10.1186/s40538-021-00224-9

Pino, J.A., Roncal, E., Rosado, A., Goire, I. (1995a). Herb oil of dill (*Anethum graveolens* L.) grown in Cuba. *J. Essent. Oil Res.* 7, 219–220. https://doi.org/10.1080/10412905.1995.9698505

Popović, V., Maksimović, L., Adamović, D., Sikora, V., Ugrenović, V., Filipović, V., Mačkić, K. (2019). Yield of biomass and essential oil of dill (*Anethum graveolens* L.) grown under irrigation. *Ratar. i Povrt.* 56, 49–55. https://doi.org/10.5937/ratpov56-19792

Radulescu, V., Popescu, M.L., Ilies, D.-C. (2010). Chemical composition of the volatile oil from different plant parts of *Anethum graveolens* L. (Umbelliferae) cultivated in Romania. *Farmacia* 58, 594–600.

Rana, V.S., Blazquez, M.A. (2014). Chemical composition of the essential oil of *Anethum graveolens* aerial parts. *J. Essent. Oil-Bearing Plants* 17, 1219–1223. https://doi.org/10.1080/0972060X.2014.894894

Rostaei, M., Fallah, S., Lorigooini, Z., Abbasi Surki, A. (2018). The effect of organic manure and chemical fertilizer on essential oil, chemical compositions and antioxidant activity of dill (*Anethum graveolens*) in sole and intercropped with soybean (*Glycine max*). *J. Clean. Prod.* 199, 18–26. https://doi.org/10.1016/j.jclepro.2018.07.141

Ruangamnart, A., Buranaphalin, S., Temsiririrkkul, R., Chuakul, W., Pratuangdejkul, J. (2015). Chemical compositions and antibacterial activity of essential oil from dill fruits (*Anethum graveolens* L.) cultivated in Thailand. *Mahidol Univ. J. Pharm. Sci.* 42, 135–143. https://doi.org/https://doi.org/10.14456/mujps.2015.17

Safikhani Nasimi, N., Adavi, Z., Mansourifar, C. (2011). Effects of different urea and vermicompost rates on yield and essential oil contents of two dill (*Anethum graveolens* L.) cultivars. *J. Plant Ecophysiol.* 11, 21–33.

Said Al Ahl, H.A.H., Sarhan, A.M.Z., Abou Dahab, M.A.D., Zeid, N.E.-S.A., Ali, M.S., Naguib, N.Y. (2015). Volatile oil composition of *Anethum graveolens* affected by harvest stage. *Int. J. Plant Sci. Ecol.* 1, 93–97.

Salman, F.A., Mutar, K.A., Alewi, Z.H. (2019). Influence of foliar application of arginine and phenylalanine on growth and essential oil content in dill (*Anethum graveolens* l.) cultivars. *Res. Crop.* 20, 826–830. https://doi.org/10.31830/2348-7542.2019.122

Santos, P.A.G., Figueiredo, A.C., Lourenço, P.M.L., Barroso, J.G., Pedro, L.G., Oliveira, M.M., Schripsema, J., Deans, S.G., Scheffer, J.J.C. (2002). Hairy root cultures of *Anethum graveolens* (dill): Establishment, growth, time-course study of their essential oil and its comparison with parent plant oils. *Biotechnol. Lett.* 24, 1031–1036. https://doi.org/10.1023/A:1015653701265

Sefidkon, F. (2001). Essential oil composition of *Anethum graveolens* L. *Iran. J. Med. Aromat. Plants Res.* 8, 45–62.

Shahmohammadi, F., Darzi, M.T., Hadi, M.H.S. (2014). Influence of compost and biofertilizer on yield and essential oil of dill (*Anethum graveolens* L.). *Int. J. Adv. Biol. Biomed. Res.* 2, 446–455.

Singh, G., Maurya, S., De Lampasona, M.P., Catalan, C. (2005). Chemical constituents, antimicrobial investigations, and antioxidative potentials of *Anethum graveolens* L. essential oil and acetone extract: Part 52. J. Food Sci. 70, 208–215. https://doi.org/10.1111/j.1365-2621.2005.tb07190.x

Singh, S., Das, S., Singh, G., Perroti, M., Schuff, C., Catalan, C.A.N. (2017). Comparative studies of chemical composition, antioxidant and antimicrobial potentials of essential oils and oleoresins obtained from seeds and leaves of *Anethum graveolens* L. *Toxicology* 03, 1000119. https://doi.org/10.4172/2476-2067.1000119

Sintim, H.Y., Burkhardt, A., Gawde, A., Cantrell, C.L., Astatkie, T., Obour, A.E., Zheljazkov, V.D., Schlegel, V. (2015). Hydrodistillation time affects dill seed essential oil yield, composition, and bioactivity. *Ind. Crops Prod.* 63, 190–196. https://doi.org/10.1016/j.indcrop.2014.09.058

Stanojević, L.P., Radulović, N.S., Djokić, T.M., Stanković, B.M., Ilić, D.P., Cakić, M.D., Nikolić, V.D. (2015). The yield, composition and hydrodistillation kinetics of the essential oil of dill seeds (*Anethi fructus*) obtained by different hydrodistillation techniques. *Ind. Crops Prod.* 65, 429–436. https://doi.org/10.1016/j.indcrop.2014.10.067

Tian, J., Ban, X., Zeng, H., He, J., Chen, Y., Wang, Y. (2012). The mechanism of antifungal action of essential oil from dill (*Anethum graveolens* l.) on *Aspergillus flavus*. *PLoS One* 7. https://doi.org/10.1371/journal.pone.0030147

Tian, J., Ban, X., Zeng, H., Huang, B., He, J., Wang, Y. (2011). *In vitro* and *in vivo* activity of essential oil from dill (*Anethum graveolens* L.) against fungal spoilage of cherry tomatoes. *Food Control* 22, 1992–1999. https://doi.org/10.1016/j.foodcont.2011.05.018

Vera, R.R., Chane-Ming, J. (1998). Chemical composition of essential oil of dill (*Anethum graveolens* L.) growing in Reunion Island. *J. Essent. Oil Res.* 10, 539–542. https://doi.org/10.1080/10412905.1998.9700965

Vokk, R., Lõugas, T., Mets, K., Kravets, M. (2011). Dill (*Anethum graveolens* L.) and parsley (*Petroselinum crispum* (Mill.) Fuss) from Estonia: Seasonal differences in essential oil composition. *Agron. Res.* 9, 515–520.

Wall, D.A., Friesen, G.H. (1986). The effect of herbicides and weeds on the yield and composition of dill (*Anethum graveolens* L.) oil. *Crop Prot.* 5, 137–142. https://doi.org/https://doi.org/10.1016/0261-2194(86)90095-5

Weisany, W. (2018). Glomus intraradices (N.C. Schenck & G.S. Sm.) C. Walker & A. Schuessle enhances nutrients uptake, chlorophyll and essential oil contents and composition in *Anethum graveolens* L. *Acta Agric. Slov.* 111, 303–313. https://doi.org/10.14720/aas.2018.111.2.06

Weisany, W., Raei, Y., Ghassemi-Golezani, K. (2016). Funneliformis mosseae alters seed essential oil content and composition of dill in intercropping with common bean. *Ind. Crops Prod.* 79, 29–38. https://doi.org/10.1016/j.indcrop.2015.10.041

Yazdani, D., Jamshidi, A.H., Rezazadeh, S., Mojab, F., Shahnazi, S. (2004). Variaion of essential oil percentage and constituent at different growth stages of dill (*Anethum graveolens* L.). *J. Med. Plants* 3, 38–41. https://doi.org/20.1001.1.2717204.2004.3.11.5.5

Yili, A., Aisa, H.A., Maksimov, V. V., Veshkurova, O.N., Salikhov, S.I. (2009). Chemical composition and antimicrobial activity of essential oil from seeds of *Anethum graveolens* growing in Uzbekistan. *Chem. Nat. Compd.* 45, 280–281. https://doi.org/10.1007/s10600-009-9275-4

Zeng, H., Tian, J., Zheng, Y., Ban, X., Zeng, J., Mao, Y., Wang, Y. (2011). *In vitro* and *in vivo* activities of essential oil from the seed of *Anethum graveolens* L. against *Candida* spp. *Evidence-based Complement. Altern. Med.* 2011, 1–8. https://doi.org/10.1155/2011/659704
