# Supplementary figures and images for: Drying temperatures affect the qualitative–quantitative variation of aromatic profiling in Anethum graveolens L. ecotypes as an industrial–medicinal–vegetable plant
Source: Front Plant Sci. 2023 May 12;14:1137840. doi: 10.3389/fpls.2023.1137840 (PMC10214840; doi:10.3389/fpls.2023.1137840)

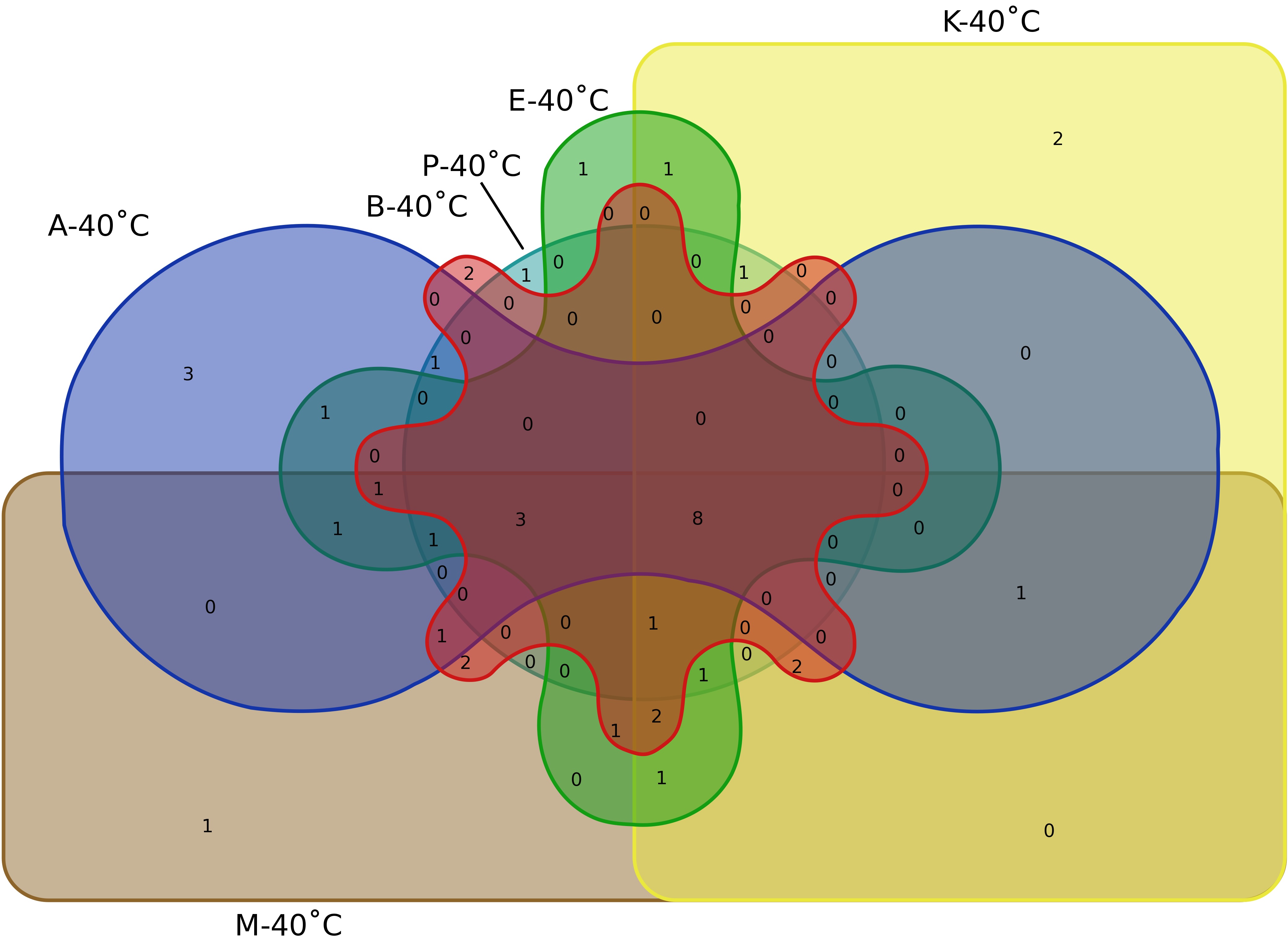

Supplement: Supplementary file 2 [file Image_1.jpeg]

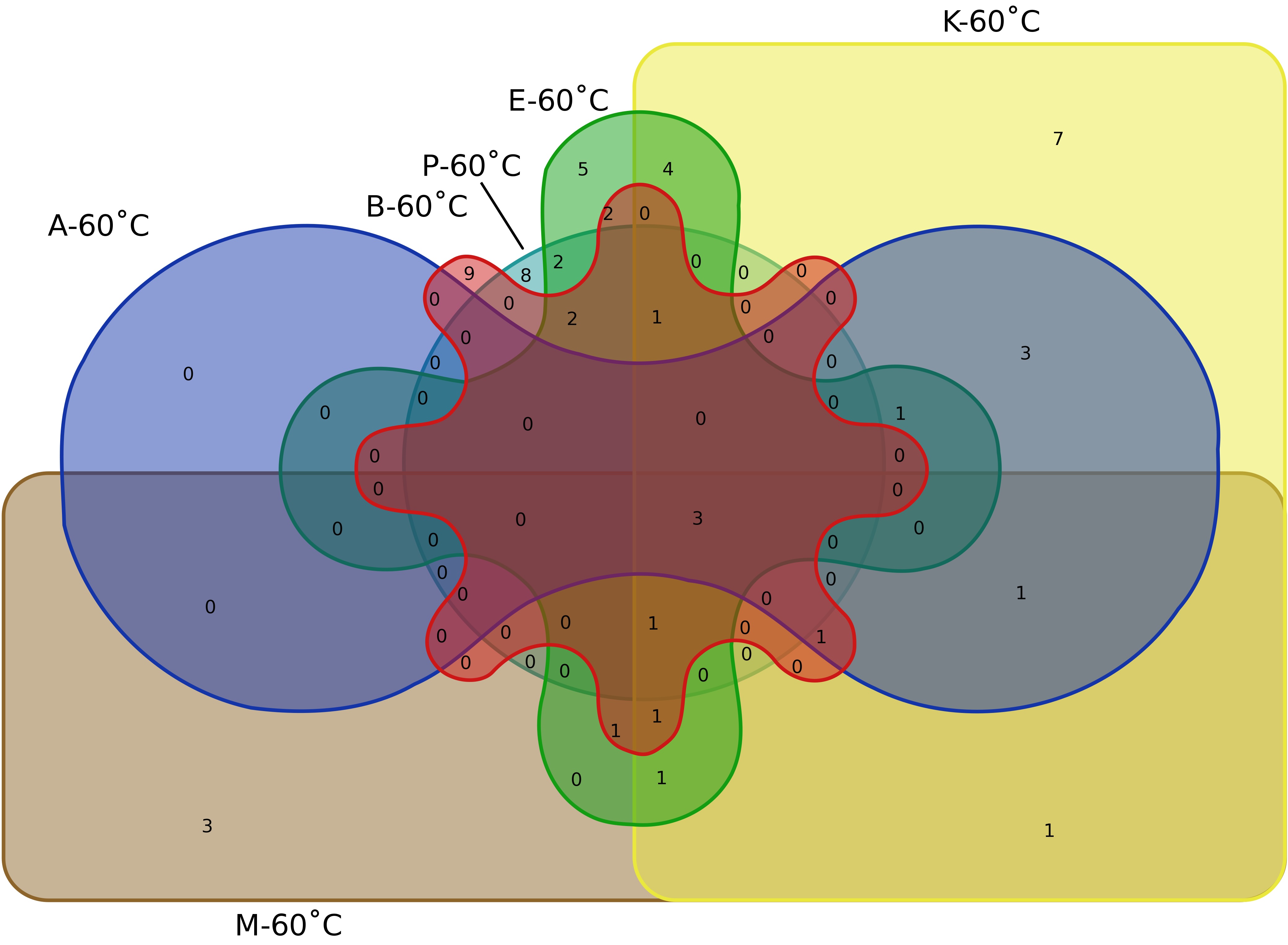

Supplement: Supplementary file 3 [file Image_2.jpeg]
